# Comparative Proteomic Analysis during the Involvement of Nitric Oxide in Hydrogen Gas-Improved Postharvest Freshness in Cut Lilies

**DOI:** 10.3390/ijms19123955

**Published:** 2018-12-09

**Authors:** Jianqiang Huo, Dengjing Huang, Jing Zhang, Hua Fang, Bo Wang, Chunlei Wang, Zhanjun Ma, Weibiao Liao

**Affiliations:** College of Horticulture, Gansu Agricultural University, Lanzhou 730070, China; huojq3061@163.com (J.H.); huangdj3032@163.com (D.H.); zhangj4517@163.com (J.Z.); fangh1610@163.com (H.F.); wangb0447@163.com (B.W.); wangchunlei@gsau.edu.cn (C.W.); mazhanjun@gsau.edu.cn (Z.M.)

**Keywords:** proteomic, postharvest freshness, ATP synthase, ATP synthase CF1 alpha subunit (chloroplast), chlorophyll fluorescence parameters, photosynthetic parameters

## Abstract

Our previous studies suggested that both hydrogen gas (H_2_) and nitric oxide (NO) could enhance the postharvest freshness of cut flowers. However, the crosstalk of H_2_ and NO during that process is unknown. Here, cut lilies (*Lilium* “Manissa”) were used to investigate the relationship between H_2_ and NO and to identify differentially accumulated proteins during postharvest freshness. The results revealed that 1% hydrogen-rich water (HRW) and 150 μM sodium nitroprusside (SNP) significantly extended the vase life and quality, while NO inhibitors suppressed the positive effects of HRW. Proteomics analysis found 50 differentially accumulated proteins in lilies leaves which were classified into seven functional categories. Among them, ATP synthase CF1 alpha subunit (chloroplast) (AtpA) was up-regulated by HRW and down-regulated by NO inhibitor. The expression level of *LlatpA* gene was consistent with the result of proteomics analysis. The positive effect of HRW and SNP on ATP synthase activity was inhibited by NO inhibitor. Meanwhile, the physiological-level analysis of chlorophyll fluorescence and photosynthetic parameters also agreed with the expression of AtpA regulated by HRW and SNP. Altogether, our results suggested that NO might be involved in H_2_-improved freshness of cut lilies, and AtpA protein may play important roles during that process.

## 1. Introduction

Hydrogen gas (H_2_), a colorless and odorless gas, is the lightest and structurally simplest gas in the world. As an important signaling molecule, H_2_ has been shown to be involved in many plant developmental processes [1]. More recently, some researchers found that H_2_ could alleviate aluminum (Al) toxicity [2], mercury (Hg) toxicity [3], and UV-A irradiation [4] by increasing the activity of antioxidant enzymes. Meanwhile, H_2_ could promote lateral root formation through nitric oxide (NO) synthesis induced by auxin [5] or in a heme oxygenase-1/carbon monoxide-dependent manner [6]. Significantly, H_2_ was also reported to play an important role in delaying senescence and maturity [7,8]. Hydrogen-rich water (HRW) treatments could prolong the shelf life of kiwifruit by regulating the antioxidant defense [7]. Our study has shown that H_2_ enhanced the vase life and postharvest quality of cut lily (*Lilium* spp.) and cut rose (*Rosa hybrid* L.) flowers through maintaining water balance and membrane stability [8]. However, the deep mechanism of H_2_ in delaying the senescence and shelf life of perishable horticultural products needs to be further investigated.

NO is a signaling molecule that interacts with other hormones and growth regulators. Recently, NO was reported to play vital roles in delaying senescence and improving the quality of horticultural products. NO inhibited the production of ethylene by modulating the expressions of some genes and proteins during postharvest of horticulture plants [9]. NO, as a preservative solution to cut flowers, can extend the vase life of cut gerbera flowers by increasing water uptake and promoting antioxidant activity [10]. Exogenous NO could also promote the vase life of cut gladiolus flowers by increasing the scavenging mechanism of reactive oxygen species (ROS) and down-regulating the expression of senescence-associated genes (SAGs) [11]. The vase life of cut carnation flowers was significantly prolonged by exogenous NO, which improved the activity of antioxidant enzymes including superoxide dismutase (SOD), peroxidase (POD), catalase (CAT), and ascorbate peroxidase (APX) [12]. Our previous study reported that NO could decrease ethylene production in cut roses by inhibiting the activity of 1-aminocyclopropane-1-carboxylate oxidase (ACO), thus promoting the vase life of cut roses [13].

As mentioned above, H_2_ and NO as exogenous gaseous signaling molecules played exceedingly positive roles in the postharvest preservation of horticultural products. The relationship between H_2_ and NO in plants has been reported in recent years. H_2_ was reported to regulate stomatal movement, which is involved in the abscisic acid (ABA) signaling cascade by promoting the generation of NO [14]. Meanwhile, Zhu et al. [15] also found that the adventitious root formation in cucumber explants induced by H_2_ was dependent on the NO pathway [15]. Furthermore, H_2_ could alleviate the Al-induced inhibition of alfalfa root elongation by inhibiting the production of NO [16]. H_2_ also was reported to be involved in auxin-induced lateral root formation, at least partially via a nitrate reductase (NR)-dependent NO synthesis [5]. Up to now, far too little attention has been paid to the crosstalk between H_2_ and NO during the postharvest preservation of horticultural plants. In this study, pharmacological approaches and comparative proteomic analysis were applied to investigate the roles of H_2_ and NO during the postharvest storage of cut lily (*Lilium* “Manissa”) flowers and to identify the differentially accumulated proteins during that process. Thus, the study offers some important insights into the protein changes in the NO–H_2_-regulated postharvest preservation of cut flowers.

## 2. Results

### 2.1. Effects of HRW, Sodium Nitroprusside (SNP), and NO Inhibitors on Vase Life

**Figure 1:**
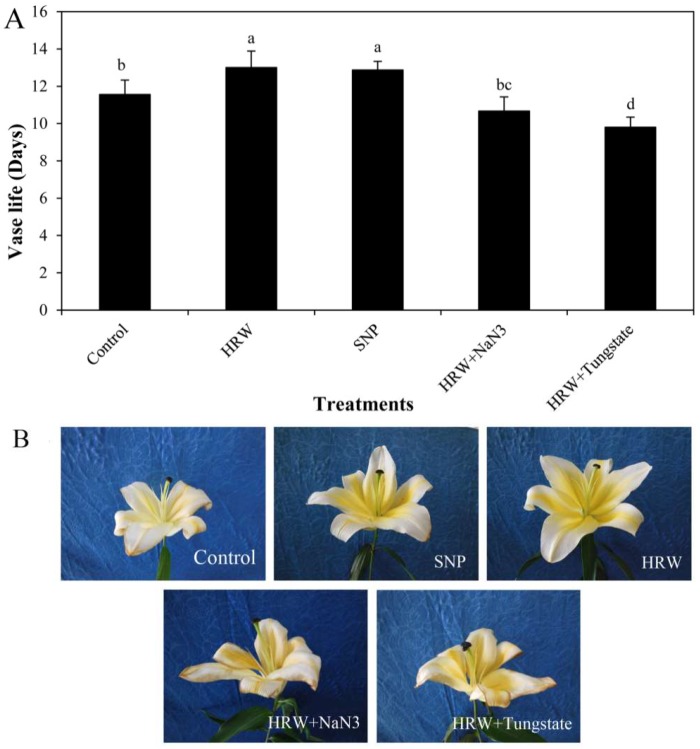
Effects of hydrogen-rich water (HRW), sodium nitroprusside (SNP), and HRW plus NaN_3_ or tungstate on the vase life of cut lily flowers. The cut flowers were placed randomly in distilled water (control), 150 μM SNP, 1% HRW, 1% HRW + 50 μM NaN_3_ and 1% HRW + 100 μM tungstate to investigate. The values of vase life (**A**) are the mean ± SE of three independent experiments. Bars with different letters illustrate significant differences (*p* < 0.05) according to Duncan’s multiple range test. Photos (**B**) were taken after 8 days of treatments.

Compared with the control (distilled water), the vase life of cut lilies was extended by applying 150 μM SNP or 1% HRW (Figure 1). However, there was no significant difference between SNP and HRW. Compared with the HRW, 1% HRW together with 50 μM NaN_3_ or 100 μM tungstate significantly decreased vase life, indicating the involvement of NO in the HRW-enhanced vase life of cut lilies (Figure 1). 

### 2.2. Effects of HRW, SNP, and NO Inhibitors on Maximum Flower Diameter and Rate of Fresh Weight Change

**Figure 2:**
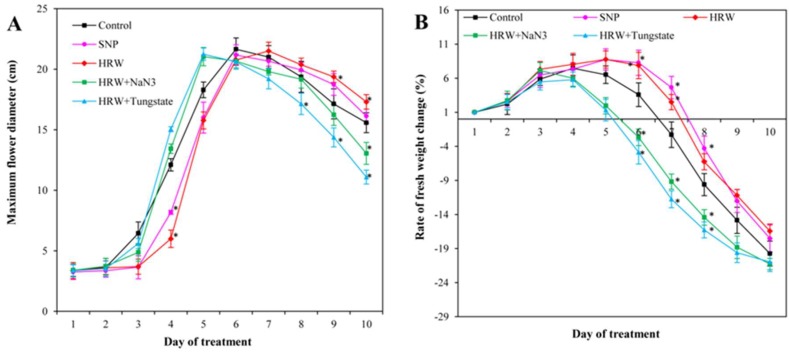
Effects of HRW, SNP, and HRW plus NaN_3_ or tungstate on flower diameter and fresh weight of the cut lilies. Maximum flower diameter (**A**) and rate of fresh weight change (**B**) were expressed as mean ± SE of three independent experiments. Asterisks indicate significant difference (*p* < 0.05 by Duncan’s multiple range test) compared to the control within the same day.

As shown in Figure 2A, the maximum value of the maximum flower diameter in the control and SNP treatment was obtained on the sixth day. The maximum value in HRW treatment appeared at the seventh day, while when HRW was applied with NaN_3_ or tungstate, the maximum values were detected on the fifth day (Figure 2A). Interestingly, various vase solutions had no effects on the maximum flower diameter, suggesting that HRW merely delayed the flowering time rather than expanding the flower diameter. 

As time passed, the rate of fresh weight change initially increased and then decreased (Figure 2B). Compared with the control, the decrease of fresh weight in HRW or SNP treatment was postponed for one day after treatment, whereas the decrease of fresh weight in HRW plus tungstate treatment significantly appeared one day in advance. The decrease of fresh weight in HRW or SNP was significantly lower than in HRW with NaN_3_ or tungstate (Figure 2B), indicating that the inhibition of endogenous NO could decrease the effect of HRW.

### 2.3. Two-Dimensional Electrophoresis Analysis and Identification of Proteins

**Figure 3:**
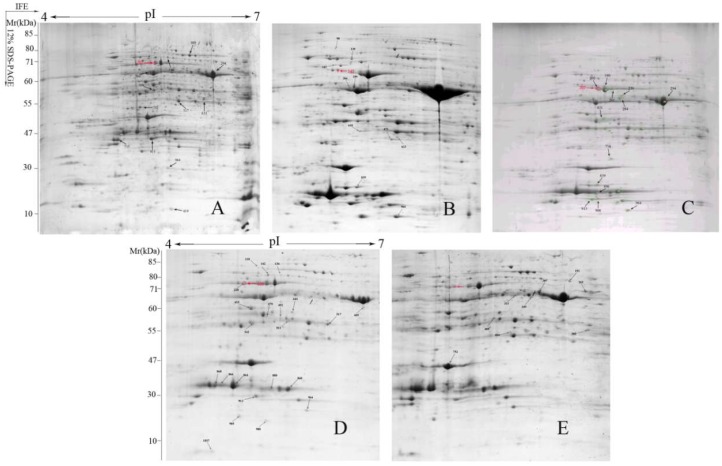
Two-dimensional electrophoresis (2-DE) image analysis of cut lily leaf proteins. Arrows indicate the 77 protein spots that were detected in control (**A**); 150 μM SNP (**B**); 1% HRW (**C**); 1% HRW + 50 μM NaN_3_ (**D**); and 1% HRW + 100 μM tungstate (**E**). The red arrows show the spots of differentially accumulated ATP synthase CF1 alpha subunit (chloroplast) (AtpA) protein under different treatments.

In the study, the differentially accumulated proteins between control and treatments (SNP, HRW, HRW + NaN_3_ or tungstate) were analyzed. In comparison of these two-dimensional electrophoresis (2-DE) gel images (Figure 3), 77 protein spots where the abundance was detected at ratios over 1.5-fold and false discovery rate (FDR) less than 5% were obtained on these images to analyze their basic information and function by 2-DE coupled to MALDI-TOF/TOF-MS. From these protein spots, 50 differentially accumulated proteins were successfully identified from the NCBI and Uniprot databases by Mascot analysis (Table 1 and Table S1). The molecular weights and isoelectric points (pIs) of identified proteins presented a different degree of variation, with molecular weights ranging from 16.70 kDa to 81.88 kDa and with pIs ranging from 4.83 to 9.35 (Table 1). Eleven protein spots were identified in the HRW treatment, while 10 spots were identified in the SNP treatment (Figure 3 and Figure S1). However, 20 or 9 protein spots were identified in HRW with NaN_3_ or tungstate, respectively. 

### 2.4. Functional Classification and Analysis of Differentially Accumulated Proteins

**Figure 4:**
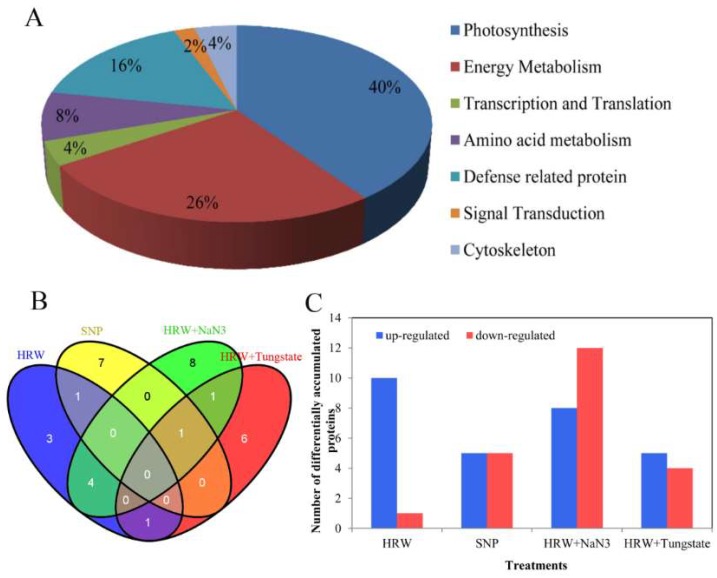

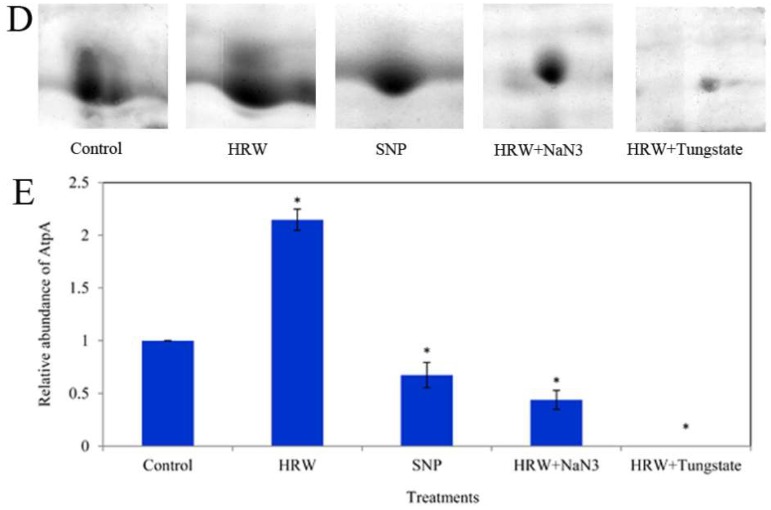
Functional classification and analysis of differentially accumulated proteins in cut lilies. Pie chart showed percentage of differentially accumulated proteins in different functional categories (**A**); Venn diagram showed the number of overlap proteins regulated by HRW, SNP, HRW + NaN_3_ or tungstate compared with the control (**B**); Column chart showed the number of up- or down-regulated proteins in comparison with the control (**C**); The 2-DE gel sections showed the magnified views of differentially accumulated spots of AtpA protein in treatments. Spot positions corresponding to AtpA protein were shown with red arrows in Figure 2 (**D**); The column chart showed the differential relative abundance patterns among HRW, SNP, HRW + NaN_3_ and HRW + tungstate treatment (**E**).

These differentially accumulated proteins were analyzed in order to classify them in terms of their biological functions according to Gene Ontology and UniProt Protein Knowledgebase. The 50 differentially accumulated proteins identified in this study were classified into seven functional categories, as shown in below: photosynthesis (40%), energy metabolism (26%), defense-related protein (16%), amino acid metabolism (8%), transcription and translation (4%), cytoskeleton (4%), and signal transduction (2%) (Figure 4A). Subsequently, the differentially accumulated proteins among HRW, SNP, HRW + NaN_3_, and HRW + tungstate treatments were analyzed. In HRW treatment, some differentially accumulated proteins that were associated with photosynthesis were detected, and many of them were increased when compared with the control. Among them, ATP synthase CF1 alpha subunit (chloroplast) (AtpA) was identified with three spots (180, 201, and 202), and all were up-regulated (Table 1). Under the SNP treatment, differentially accumulated proteins including FtsH-like protein Pftf (spot 182), trypsin-like serine protease (spot 618), and putative L-ascorbate peroxidase 2, cytosolic-like (spot 839) were observed. The expression of these three proteins related to defense was higher than that of the control. In HRW + NaN_3_ treatment, two spots were identified as AtpA, and they were down-regulated when compared with the control. The expression of proteins related with defense was significantly decreased, such as Type II peroxiredoxin (spot 1037) and pathogenesis-related protein 10 (spot 1060). Under the treatment with HRW plus tungstate, photosystem II oxygen evolving complex protein 1 precursor (spot 752) belonged to photosynthesis, and malic enzyme (spot 192) associated with energy metabolism were all down-regulated in comparison with the control (Table 1). There were four differentially accumulated proteins overlapping between HRW and HRW + NaN_3_ (Figure 4B). After statistical analysis, 28 proteins in total were up-regulated while 22 proteins were down-regulated (Table 1). Among them, the number of up-regulated proteins was significantly higher in HRW than in HRW + NaN_3_ or tungstate (Figure 4C). However, the number of down-regulated proteins was lower in HRW than in HRW + NaN_3_ or tungstate (Figure 4C). Thus, exogenous H_2_ could up-regulated some proteins during postharvest storage of cut lilies.

Interestingly, among them, only AtpA was up-regulated in HRW treatment and down-regulated in the HRW with NaN_3_ treatment. Therefore, AtpA was selected to further investigate. The magnified views of 2-DE image showed the differential accumulation of AtpA protein in treatments of the control, HRW, HRW + NaN_3_ and HRW + tungstate (Figure 4D). The relative abundance of AtpA protein has significant difference in 4 treatments (Figure 4E). The relative abundance of AtpA in HRW treatment was significantly higher than that in the control, while the relative abundance of AtpA was less in SNP than in the control. However, the relative abundance of AtpA protein in HRW + NaN_3_ treatment was significantly less than that in HRW treatment, and the relative abundance of AtpA was not detected in HRW + tungstate (Figure 4E), which may be caused by too low differential accumulation of AtpA in HRW + tungstate. Taken together, H_2_ could enhance the expression of AtpA protein, and the inhibitors of NO (NaN_3_ and tungstate) may have inhibited the effect of H_2_ on the expression of AtpA protein.

### 2.5. Relative Expression of LlatpA Gene and the Activity of ATP Synthase (ATPase)

**Figure 5:**
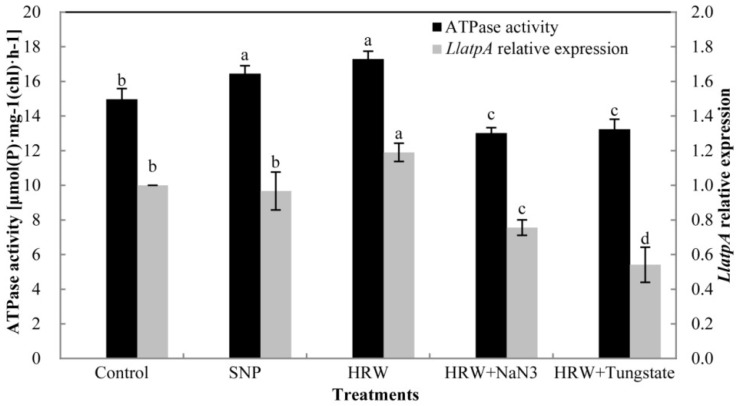
Effects of SNP, HRW, and HRW in combination with NaN_3_ or tungstate on *LlatpA* gene expression and ATP synthase (ATPase) activity. Values of relative expression of *LlatpA* gene and activity of ATP synthase are the mean ± SE of three independent experiments with three repeats for each. Bars with different letters illustrate significant differences (*p* < 0.05) according to Duncan’s multiple range test.

The qRT-PCR analysis revealed that the relative expression of the *LlatpA* gene was significantly higher in HRW treatment than in the control (Figure 5). There was no significant difference in the gene expression between the control and SNP treatment. In comparison with HRW treatment, the relative expression of *LlatpA* gene was significantly inhabited by HRW plus NaN_3_ or tungstate. As shown in Figure 5, compared with control, the activity of ATPase was remarkably enhanced by SNP or HRW. However, the activity of ATPase in HRW plus NaN_3_ or tungstate treatment was decreased in comparison with HRW treatment.

### 2.6. Chlorophyll Fluorescence and Photosynthetic Parameters

**Figure 6:**
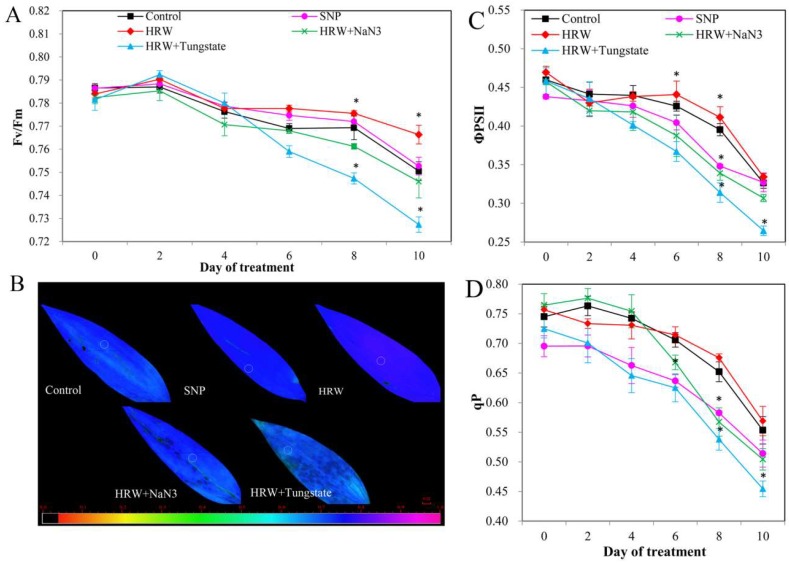
Effects of HRW, SNP, and HRW plus NaN_3_ or tungstate on chlorophyll fluorescence parameters. Values of the Fv/Fm (maximum quantum yield of PSII photochemistry) (**A**); effective quantum yield of PSII (ΦPSII) (**C**); and photochemical quenching (qP) (**D**) are the mean ± SE of three independent experiments with three repeats for each. Fluorescent images (**B**) are given in colors that represent the absolute values of the ratio ranging from 0 (black) to 1.0 (purple) and were taken on the 8th day of treatment. Asterisks indicate significant difference (*p* < 0.05 by Duncan’s multiple range test) compared to the control within the same day.

The chlorophyll fluorescence parameters analysis result is shown in Figure 6. After 6 days of treatment, the maximum quantum yield of photosystem Ⅱcomplex (PSII) photochemistry (Fv/Fm) in HRW or SNP groups was higher than that in the control, whereas HRW in combination with NaN_3_ or tungstate significantly inhibited the positive effects of HRW (Figure 6A,B). After treatment for 6 days, when compared with the control, the effective quantum yield of PSII (ΦPSII) and photochemical quenching (qP) were increased in HRW treatment. However, significantly decreased ΦPSII and qP appeared in the SNP treatment group. ΦPSII and qP in HRW plus NaN_3_ or tungstate treatment were significantly decreased in comparison with those in the HRW treatment (Figure 6C,D).

**Figure 7:**
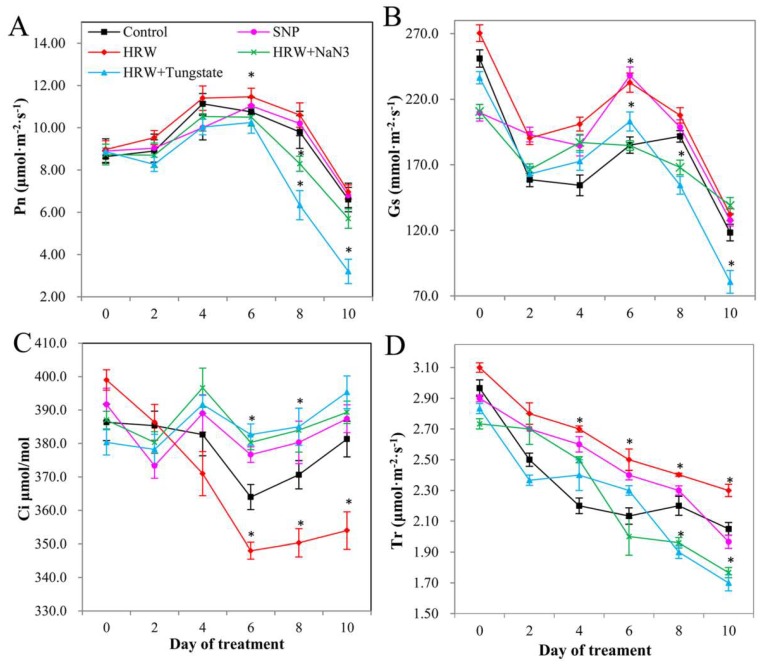
Effects of HRW, SNP, and HRW plus NaN_3_ or tungstate on photosynthetic parameters. Values of net photosynthetic rate (Pn) (**A**), stomatal conductance (Gs) (**B**), the intercellular CO_2_ concentration (Ci) (**C**), and transpiration rate (Tr) (**D**) are the mean ± SE of three independent experiments with three repeats for each. Asterisks indicate significant difference (*p* < 0.05 by Duncan’s multiple range test) compared to the control within the same day.

A downward trend was also observed in the net photosynthesis rate (Pn; Figure 7A) and stomatal conductance (Gs; Figure 7B) after 6 days of treatment. In contrast, intercellular CO_2_ concentration (Ci; Figure 7C) was slightly increased after treatment for 6 days. Transpiration rate (Tr) showed a tendency to decrease during the experiment (Figure 7D). Pn, Gs, and Tr were significantly increased by HRW or SNP in comparison with those in the control group. HRW plus NaN_3_ or tungstate treatment significantly decreased Pn, Gs, and Tr when compared with HRW treatment (Figure 7A,B,D). Interestingly, Ci in HRW treatment was lower than that in the control, whereas Ci in HRW together with NaN_3_ or tungstate treatment was higher than that in HRW treatment (Figure 7C). 

## 3. Discussion

H_2_ is considered as a novel signaling molecule involved in plant developmental and physiological processes [17]. Our previous studies showed that exogenous H_2_ could enhance adventitious root development in marigold [18]. In addition, the shelf life of kiwifruit was prolonged by H_2_ by decreasing ethylene biosynthesis [19] and reducing oxidative damage [7]. In this study, H_2_ delayed the flowering time of cut lilies in the preservation process. H_2_ improved the vase life of cut lilies by maintaining the fresh weight of cut lilies. The results were consistent with those of Ren et al. (2017) [8], who showed that exogenous H_2_ enhanced the vase life of cut flowers by maintaining suitable water balance. In the current study, the vase life of cut lily was also enhanced by SNP treatment, suggesting that exogenous NO may play an important role in extending vase life. The vase life of cut gerbera was significantly extended by exogenous NO [10]. Exogenous NO also could delay petal wilting in cut carnation flowers by maintaining water metabolism and antioxidant enzyme activity [12]. In our study, NaH_3_ and tungstate—inhibitors of nitrate reductase (NR) that can inhibit the reduction of nitrate to nitrite and further inhibit the production of NO—were used to investigate whether NO participates in H_2_-regulated postharvest preservation. NO inhibitors NaN_3_ or tungstate depressed the positive effects of H_2_ on the vase life, the maximum flower diameter, and fresh weight of cut lilies, suggesting that NO played vital roles in H_2_-induced cut flowers freshness. Our previous studies revealed that H_2_ increased NO generation through regulated NR and NOS activity to induce the formation of adventitious root in cucumber [20]. H_2_, as a mediator, activated cell cycle by NO pathway during adventitious root formation [15]. Additionally, H_2_ was also involved in auxin-induced lateral root formation via an NR-dependent NO synthesis [5]. Here, for the first time, the involvement of NO in hydrogen gas-improved vase life in cut flowers was reported.

Two-dimensional electrophoresis (2-DE)-based proteomics analysis has been applied in plant proteomic research. Here, the results of the comparative proteomic analysis showed that 50 differentially accumulated proteins were successfully identified by Mascot analysis in cut lily leaves. Among them, 28 proteins were up-regulated while 22 proteins were down-regulated. Exogenous H_2_ could increase the number of up-regulated proteins, while inhibitors of NO increased the number of down-regulated proteins. In chrysanthemum cuttings during adventitious root formation, 42 differentially accumulated protein spots were successfully matched to NCBI database entries [21]. In cut rose flowers, 103 proteins were obtained, and these proteins were involved in plant growth regulators, natural resistance, protein metabolism, and methionine synthesis [22]. In the current study, the 50 differentially accumulated proteins were involved in photosynthesis, energy metabolism, defense, amino acid metabolism, etc. In a *Medicago sativa* cadmium resistance study, the proteins related to photosynthesis were not detected in H_2_ treatment [23]. However, we found that the proteins involved in photosynthesis showed a high expression level in H_2_ treatment. This may be caused by different experimental materials and conditions. The proteins related to the stress response and defense changed significantly after NO treatment in the processes of peach fruit ripening, such as glutathione S-transferase (GST) and ascorbate peroxidase (APX) [24]. Simultaneously, we found that the expression of proteins associated with defense were up-regulated by exogenous NO in the cut lilies during preservation. This suggested that NO could promote the expression of proteins related to defense. The proteins related to energy metabolism were decreased during strawberry fruit ripening [25]. In litchi pulp, malate dehydrogenase (related to energy metabolism) was down-regulated in the later storage period [26]. In this study, the proteins related to energy metabolism were down-regulated in HRW plus tungstate, suggesting that NO played an important role in the proteins’ expression, regulated by H_2_. Thus, H_2_ and NO could regulate the expression of proteins related to photosynthesis, defense, and energy metabolism while delaying the senescence of cut lilies.

ATP synthase CF1 alpha subunit (AtpA) protein is a key enzyme for the chloroplast thylakoid membranes, and plays a vital role in synthesizing ATP from ADP and phosphate [27]. ATP synthase CF1 α-subunit was obtained and showed an initial increase and then a decrease in *Kandelia candel* under salt stress [28]. The expression of ATP synthase CF1 α-subunit was decreased in the treatment of MAP kinase kinase (MEK) inhibitor in *Chlamydomonas reinhardtii* [29]. In this study, we revealed that the differential relative abundance of AtpA protein was significantly different between experimental treatments. H_2_ could up-regulate the expression of AtpA protein during postharvest freshness of cut lilies, while the accumulation of AtpA protein was not significantly up-regulated by NO. Interestingly, AtpA protein was down-regulated in HRW + NaN_3_, but no accumulation spots of AtpA protein were detected in HRW + tungstate treatment. This may be because the expression of AtpA was too low (abundance ≤ 1.5-fold) to detect in the HRW + tungstate group. This suggested that the positive effect of H_2_ on the expression of AtpA protein was inhibited by the inhibitors of NO. The positive roles of H_2_ on polyphenol oxidase activity were impaired by cPTIO (NO scavenger), L-NAME (NO synthase enzyme inhibitor), and NaN_3_ [22]. H_2_-promoted NO accumulation and stomata closure were greatly prevented by L-NAME or tungstate [14]. The above results suggest that the positive effects of H_2_ were reversed when the generation of NO was blocked by an inhibitor or scavenger. Thus, it may be that H_2_ at least partially played its positive roles through endogenous NO. In the study, the relative expression of *LlatpA* gene and the activity of ATPase were determined in order to further investigate the expression of AtpA protein at the transcriptional and biochemical levels. ATPase is embedded in the same coupling membrane, and is composed of several subunits, including an alpha subunit. The a-subunit is composed of five transmembrane helices (TMHs), including a four-helix bundle [30]. The prerequisite of ATPase exerting its proton-driven role is intersubunit mobility. Thus, the CF1 a-subunit plays an important role in ATPase. Additionally, ATPase is a key thylakoid membrane protein encoded by the *atpA* gene of the chloroplast genome [31]. In our study, the RT-qPCR results showed that exogenous H_2_ could increase the expression of the *LlatpA* gene. The expression of the *atpA* gene of cucumber was increased by exogenous putrescine in salt stress [32]. H_2_ could get into soluble spinach chloroplast to activate ATPase by exchanging into internal parts of the molecule on energized membranes [33]. Simultaneously, the change in the expression of the *atpA* gene was positively related to ATPase activity at the transcriptional level under low temperature conditions [34]. Meanwhile, we revealed that the activity of ATPase was also promoted by H_2_, which was positively related to the relative expression of the *LlatpA* gene. In another study, NO was found to play a vital role in stimulating H^+^-ATPase activity during the early stages of maize lateral root development [35]. Exogenous NO could alleviate the inhibition of H^+^-ATPase in plasma membrane or tonoplast which was induced by CuCl_2_ [36]. In the present study, exogenous NO could also enhance the activity of ATPase. However, the positive effects of H_2_ on the *LlatpA* gene and ATPase were inhibited by inhibitors of NO. The transcription levels of the cyclin-dependent kinase B decreased when H_2_ was used together with cPTIO, L-NAME, and NaN_3_, respectively [15]. It was suggested that NO may act as a signaling molecule involved in H_2_ to increase the expression of the *LlatpA* gene and the activity of ATPase. All of these results were consistent with the expression of AtpA protein. Taken together, H_2_ may play its positive role in the expression of the *LlatpA* gene and the activity of ATPase by regulating endogenous NO. 

The results of transcriptional level and biochemical level analysis were consistent with the results of AtpA protein expression, suggesting that the involvement of NO in the H_2_-promoted vase life of cut lilies may be through regulation of the expression of AtpA protein. Since AtpA is a protein related to photosynthesis, and ATPase plays significant roles in photosynthesis-dependent membrane hyperpolarization and energy transfer [37], in the next study, the chlorophyll fluorescence parameters and photosynthetic parameters were determined to further validate the effects of the ATP protein on NO and H_2_ co-regulated postharvest preservation at the physiological level. We found that exogenous H_2_ could increase the value of Fv/Fm, ΦPSII, and qP. A previous study also found that exogenous H_2_ could significantly alleviate high light induced-damage to PSII [38]. In this study, proteomics analysis suggested that the expression of AtpA was up-regulated by H_2_. Therefore, H_2_ played positive roles in enhancing the light energy conversion efficiency of PSII, possibly by regulating the expression of AtpA protein. Exogenous NO could alleviate paraquat-induced decline of Fv/Fm [39]. Exogenous NO also could significantly increase Fv/Fm, and thereby the toxic effects of arsenic (As) on photosynthesis were alleviated in *Luffa* seedlings [40]. The exogenous NO could decrease qP to inhibit the electron transport rate (ETR) [41]. In this study, the ratio of Fv/Fm was increased by NO. However, exogenous NO did not significantly change the values of ΦPSII and qP, suggesting that NO had no obvious role in the capture and distribution of light energy. Exogenous NO could remarkably alleviate the inhibition of Fv/Fm induced by chilling stress, while inhibitors of NO could reduce Fv/Fm, ФPSII, and qP [42]. Our data also revealed that the positive effects of H_2_ on Fv/Fm, ΦPSII, and qP were inhibited by inhibitors of NO. This result was consistent with the result that inhibitors of NO inhibited the positive effects of H_2_ on the expression of the AtpA protein. Thus, the involvement of NO in the H_2_-regulated electron transport of PSII may be by regulating AtpA protein. Photosynthesis leads to the storage of solar energy in organic compounds. As a key enzyme related to photosynthesis, AtpA protein may affect photosynthesis efficiency [43]. Here, the results of photosynthetic analysis showed that Pn and Gs were increased by exogenous H_2_ or NO, but the value of Ci decreased. The above results were consistent with the proteomics analysis, indicating that H_2_ enhanced photosynthesis by regulating the expression of AtpA protein. Exogenous H_2_ could also increase the Pn in a concentration-dependent manner in maize seedlings [38]. Exogenous NO effectively inhibited the decrease in Pn as a result of non-stomatal factors under acid rain stress [44]. These results suggested that NO and H_2_ played a positive role in improving the photosynthetic performance of cut lily leaves. Chen et al. (2014) [45] reported that the Pn of transgenic and wild-type rice plants was significantly increased by NO, while the effect of NO on the Pn was inhibited by an NO scavenger. The positive effect of H_2_ on alleviating the Al-induced inhibition of alfalfa root growth was inhibited by cPTIO (a scavenger of NO) and tungstate [16]. In the present study, the effect of H_2_ on Pn and Gs were decreased by inhibitors of NO. However, the Ci was increased when the roles of H_2_ were inhibited by NO inhibitors. This may be due to the positive relationship between intercellular CO_2_ concentration (Ci) and net photosynthetic rate (Pn) under stomatal opening. Proteomics analysis also showed that the positive effect of H_2_ on improving the expression of AtpA protein was inhibited by NO inhibitors. Therefore, the involvement of NO in H_2_-regulated photosynthesis may be through regulating the expression of AtpA protein. 

**Figure 8:**
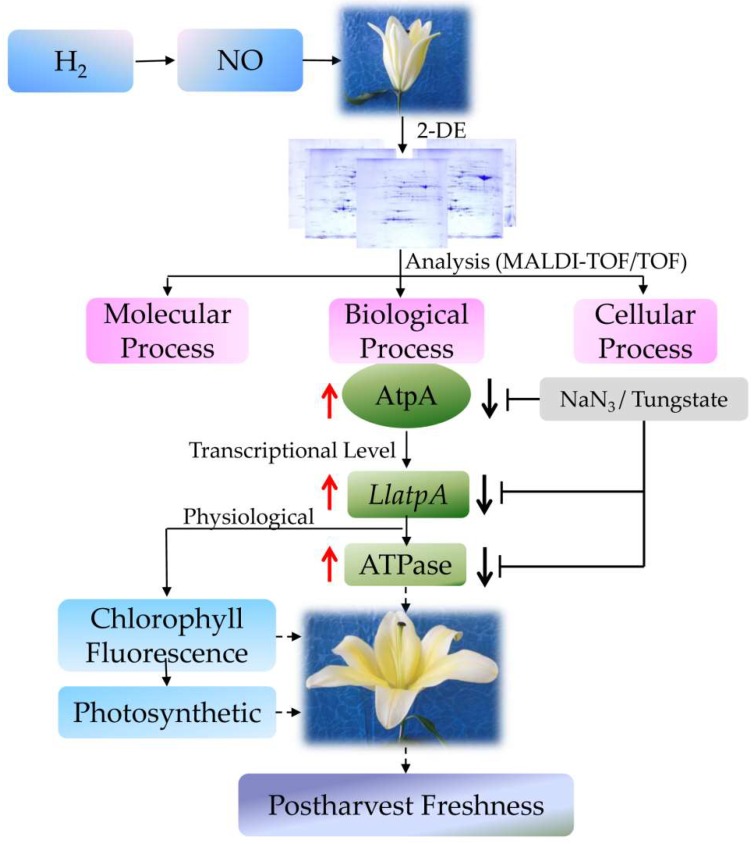
Schematic diagram of key proteins during nitric oxide–hydrogen gas-improved postharvest freshness in cut lily by comparative proteomic analysis.

In conclusion, exogenous H_2_ or NO significantly promoted the vase life and quality of cut lilies, and NO might play an important role in the H_2_-improved postharvest freshness of cut lilies. Additionally, H_2_ also significantly regulated the expression of AtpA protein and the activity ATPase, as well as photosynthesis in the postharvest freshness of cut lilies (Figure 8). Interestingly, NO may be involved in this process. Collectively, our results also revealed that NO was involved in the H_2_-enhanced shelf-life and quality of cut lilies, possibly through regulating the expression of the photosynthesis-related AtpA.

## 4. Materials and Methods

### 4.1. Plant Material and Treatments

Cut lily (*Lilium* “Manissa”) flowers with a single green bud and similar flowering degree were obtained from a commercial grower (Qianxi Florist, Lanzhou, China) and transferred rapidly to the laboratory. Flowers were held in water for 12 h and then cut under water to a length of 45 cm and every flower having five leaves on the top was kept to provide homogenous samples. Finally, the flowers were inserted into 1 L of treatment solution: distilled water (the control), 1% hydrogen-rich water (HRW, the preparation of HRW was based on the method of Zhu et al. [20]), 150 μM sodium nitroprusside (SNP, a donor of NO), 1% HRW plus NO inhibitors 50 μM sodium azide (NaN_3_) or 100 μM tungstate. The above chemicals were purchased from Sigma-Aldrich (St Louis, MO, USA) except for tungstate (tungstate was provided from Shanghai Zhaoyun chemical Co., Ltd., Shanghai, China). The treatment solution was exchanged every day at regulating time. Furthermore, each treatment was conducted in three replicates, with each replication including five cut flowers. The laboratory was maintained at 25 ± 3 °C, 60% ± 5% relative humidity, and 15 μmol·m^−2^·s^−1^ photons irradiance.

### 4.2. Determination of Vase Life and Maximum Flower Diameter 

The vase life of cut lily flowers was determined according to time beginning on the first day when flowers were inserted in the vase solution. The vase life was considered to the termination when the flower was wilted. A Vernier caliper was used to measure the maximum flower diameter, which is the maximum distance between buds and petals. The cross method was conducted to measure the maximum flower diameter. After treatment, the maximum flower diameters were measured and recorded every day.

### 4.3. Determination of the Rate of Fresh Weight Change 

The fresh weight of each flower before treatment was measured using an electronic balance and recorded as *W*_0_ (*W*_0_ is the fresh weight of the cut lilies at the first day). Then, the water at the base of the flower stem was blotted with filter paper and its fresh weight was measured. The value was recorded as *W*_d_ (*W*_d_ is the fresh weight of the cut lilies at *d* = 1, 2, 3… days). The rate of fresh weight change was calculated according to the formula: [(*W*_d_ − *W*_0_)/*W*_0_] × 100.

### 4.4. Protein Extraction

Three biological replicates were performed in the comparative proteomic analysis. The sample of 2 g cut lily leaves was used to extract protein in the eighth day of treatment. Each sample was ground to a fine powder with 0.04 g polyvingypyrrolidone (PVPP) in liquid nitrogen in a pre-cooled mortar. The powders were transferred into six 2 mL tubes. To each tube was added ice-cold trichloroacetic acid (TCA)/acetone (containing 10% (*v*/*v*) TCA and 0.07% (*v*/*v*) β-mercaptoethanol (β-ME)). Samples were mixed with a vortex and placed in a freezer at −20 °C overnight. The next day, the pellet was centrifuged at 20,000× *g* for 30 min at 4 °C. Two milliliters of 100% (*v*/*v*) pre-cooled acetone (containing 0.07% β-ME) was added, mixed with a vortex and placed at −20 °C for 1 h, centrifuging at 20,000× *g* for 20 min at 4 °C and discarding the supernatant. Then, the step was repeated again. Two milliliters of 80% (*v*/*v*) pre-cooled acetone (containing 0.07% β-ME) was added, mixed by vortex and placed at −20 °C for 30 min, centrifuged at 20,000× *g* for 15 min at 4 °C, and the supernatant was discarded. Then, the step was repeated twice. The pellet was placed in a 2 mL centrifuge tube precooled with liquid nitrogen and placed in an in situ ordinary type freeze dryer (Scientz-10ND, Ningbo Xinzhi Biotechnology Co., Ltd., Ningbo, China) to dry to a white powder. A certain amount of 7 M protein lysate containing DL-dithiothreitol (DTT) was added and cracked at room temperature for 2 h, mixed by vortex one time per 30 min during cracking and centrifuged at 20,000× *g* for 30 min at 4 °C. The supernatant was the total protein of lily leaves. The resulting protein was stored at −80 °C for next use. The protein concentration was determined according to the method described by Bradford assay (Bio-Rad, Hercules, CA, USA). 

### 4.5. Two-Dimensional Electrophoresis (2-DE) and Gel Image Analysis

For two-dimensional electrophoresis (2-DE), a total of 0.8 mg protein was first subjected to isoelectric focusing (IEF) and separated by two-dimensional SDS-PAGE. First-dimension IEF was done using pH 4–7 NL IPG strips (ReadyStrip, 17 cm, BioRad, USA). The strips were rehydrated in a rehydration solution (7 M urea, 2 M thiourea, 4% CHAPS, 65 mM DTT, 0.2% (*w*/*v*) Bio-Lyte, and 0.001% bromophenol blue) containing the protein sample for 12 h at room temperature. IEF was conducted in a Protean^®^ IEF Cell (Bio-Rad) at 20 °C, and the voltage was set as 50 V for 14 h, 250 V for 3 h, 1000 V for 5 h, 9000 V for 5 h, and 9000 V until maximum 90,000 Vh, then under 500 V run for 24 h. After first-dimension IEF, the strips were equilibrated instantly for 15 min in equilibration buffer I (6 M urea, 2% (*w*/*v*) SDS, 0.375 M Tris-HCl (pH 8.8), 20% (*v*/*v*) glycerol, 2% (*w*/*v*) DTT). Then, 5 mL equilibration buffer II (6 M urea, 2% (*w*/*v*) SDS, 0.375 M Tris-HCl (pH 8.8), 20% (*v*/*v*) glycerol, 2.5% (*w*/*v*) iodoacetamide) was added and incubated for 15 min. Second dimension SDS-PAGE was conducted in 12% (*v*/*v*) polyacrylamide-SDS gel (Protean^®^ Plus Dodeca Cell, Bio-Rad). After electrophoresis, the gels were stained with Coomassie Brilliant Blue (Bio-Rad). Stained 2-DE gels were scanned with a GS-800 Calibrated Densitometer (Bio-Rad), and data were analyzed by PDQuest software version 8.0 (Bio-Rad) as described by the manufacturer. The spots were automatically detected by the software and then subjected to careful manual editing and confirmation. Each spot of the standard gel accorded the following criteria: it was present in at least two of the three gels and was qualitatively consistent in size and shape in the replicate gels. The relative volume of each spot was assumed to represent the expression level of its protein. The volume of each well-separated spot was compared between control and different treatments to identify differentially accumulated protein spots. A spot abundance ratio of greater than 1.5 (*p* < 0.05) (a spot present uniquely or present in two-fold abundance in one sample relative to the other) was used as the threshold for a protein being differentially accumulated in subsequent studies. Every treatment was done with three biological replicates.

### 4.6. Protein Identification and Database Searching

Protein spots that appeared as differentially expressed between the control and treatment samples were excised from the gels and digested with trypsin (Promega, Madison, WI, USA), based upon the procedure described by Liu et al. [46]. MS and tandem mass spectrometry (MS/MS) data for protein identification were obtained by using a matrix-assisted laser desorption ionization time-of-flight (MALDI-TOF/TOF) instrument (4800 Plus MALDI TOF/TOFTM Analyzer; AB SCIEX, Framingham, MA, USA), as previously described by Sheffield et al. [47]. The MS/MS spectra searches were submitted to the NCBI database (http://www.ncbi.nlm.nih.gov) downloaded on 9 January 2017 (15,653 sequences) and Uniprot database (http://www.uniprot.org/) downloaded on 21 September 2016 (3,887,742 sequences) to identify proteins in the MASCOT (version 2.2, Matrix Science, London, UK) search engine using the following search parameters: MS tolerance of 50 ppm, MS/MS tolerance of 0.5 Da, peptide molecular mass ranging from 400 to 4000 Da, with one missing cleavage site, fixed modifications of carbamidomethyl (Cys) and variable modifications of oxidation (Met). The Percolator algorithm was used to estimate the false discovery rate (FDR) based on *p*-value, and only peptides at the 99% confidence level were counted as the identified protein. Proteins were considered as identified when one protein had to contain at least two peptides and when average of fold change was ≥1.5 in the experimentally treated groups compared to the control group.

### 4.7. Quantitative RT-PCR (qRT-PCR) Analyses

After 8 days of treatment, 1 g of the cut lily leaves was ground to a powder in liquid nitrogen and the total RNA was abstracted using TaKaRa MiniBEST plant RNA extraction kit (Takara Bio Inc, Kusatsu, Shiga, Japan) according to the manufacturer’s instructions. For these samples, 1 μg of total RNA was converted to cDNA using PrimeScript RT Master Mix kit (Takara Bio Inc, Kusatsu, Shiga, Japan) according to the manufacturer’s instructions. Quantitative RT-PCR was conducted with SYBR^®^ Premix Ex Taq^TM^ II (Takara Bio Inc, Kusatsu, Shiga, Japan) and LightCycler® 96 (Roche Applied Science, Mannheim, Germany) automated PCR system using two-step cycling conditions of 95 °C pre-degeneration for 30 s, followed by 40 cycles of 95 °C for 5 s, and then annealing at 60 °C for 20 s. The reaction mixture (20 μL) contained 1 μL of cDNA solution and primers at a concentration of 10 μM each. The lily gene *actin* (JX826390) was used as a reference for calculating relative transcript abundance. The primers of *actin* were: forward (5′-TGCTGGATTCTGGTGATGGT-3′) and reverse (5′-TCCCGTTCAGCTGTAGTTGT-3′). The CDS of ATP synthase CF1 alpha subunit (AtpA) was acquired according to accession no. by NCBI. The *atpA* gene *Lilium* “Manissa” was named the *LlatpA* gene. The *LlatpA* gene-specific primers were designed based on the cDNA sequences. The primers of *LlatpA* were: forward (5′-AAGCTTGTGCCTGTTTGGAG-3′) and reverse (5′-AACGGCAGATTCACCTGCTA-3′). The method of comparative Ct (2^−ΔΔ*Ct*^) [48] was used to calibrate the relative quantification of RNA expression. Each sample was set three biological replicates.

### 4.8. Determination of ATP Synthase Activity 

A 1 g sample of the cut lily leaves was ground with a crude enzyme extracting solution (2.75 mL β-mercaptoethanol and 0.688 g EDTA-Na_2_, volume fixed to 1 L using 20 mmol/L Tris-HCl) to form a slurry. The slurry was filtered through four layers of gauze. Then, 1.5 mL of the filtrate was centrifuged at a speed of 12,000× *g* at 4 °C for 10 min. Subsequently, 100 μL supernatant was added in 200 μL of the reaction solution (0.5448 g ATP-Na and 0.6517 g MgCl_2_·6H_2_O were fixed to a volume of 300 mL using a 20 mmol/L maleic acid buffer) and incubated for 30 min at 38 °C. The reaction was terminated by 200 μL TCA. Finally, 1.5 mL phosphorus reagent (6 M H_2_SO_4_: distilled water: 2.5% ammonium molybdate aqueous solution: ascorbic acid = 1:2:1:1) was added to the reaction to enact a color reaction for 20 min at 45 °C. After finishing the color reaction, 3 mL distilled water was added to measure the OD value at 660 nm using a UV spectrophotometer (UV-2800A, Unico^®^ (Shanghai) Instrument Co., Ltd., Shanghai, China). The inorganic phosphorus content was calculated according to the OD_660_ and the standard curve that prepared with KH_2_PO_3_ at different concentrations was: *y* = 8.235*x* − 0.0859, *R*² = 0.9984. ATPase activity was calculated and the unit was μmol (Pi) × (mg (chl) h)^−1^).

### 4.9. Determination of Chlorophyll Fluorescence and Photosynthetic Parameters

Chlorophyll fluorescence parameters were investigated using an Imaging-PAM Chlorophyll Fluorometer (Walz, Effeltrich, Germany) at 2, 4, 6, 8, and 10 days after treatment. Before measurement, the cut lily leaves were kept in darkness for 30 min to allow all reaction centers to open. The maximum quantum yield of PSII (Fv/Fm = (Fm − Fo)/Fm) and the effective quantum yield of PSII (ФPSII) [ФPSII = (Fm’ − Fs)/Fm’] was calculated according to Genty et al. [49]. Photochemical quenching (qP) [qP = (Fm’ − Fs)/(Fm’ − Fo’)] was calculated according to Van Kooten and Snel (1990) [50]. Photosynthetic parameters were measured using a CIRAS-2 Portable Photosynthesis and Chlorophyll Fluorescence System (PP Systems Ltd., Hitchin, Herts, UK) at a photon irradiance of 1500 Lmol m^−2^ s^−1^. Three plants in each treatment were randomly selected for gas exchange measurement at 9:00 to 11:00 a.m. on a sunny morning at 2, 4, 6, 8, and 10 days after treatment. Net photosynthetic rate (Pn), transpiration rate (Tr), stomatal conductance (Gs), and intercellular CO_2_ concentration (Ci) were recorded. 

### 4.10. Statistical Analysis

Values are means ± SE of three various experiments with three replicated measurements. Multiple comparisons were performed using Duncan’s multiple range test to determine the significance of the results between different treatments at the *p* < 0.05 level. The data analysis was conducted using the software SPSS 22.0 (SPSS Inc., Chicago, IL, USA). 

## Figures and Tables

**Table 1:** Identification and analysis of proteins in leaves of cut lily after HRW, SNP, and HRW plus NaN_3_ or tungstate treatment.

Spot No.	Protein Name	Species	Accession No.	Expressed MW (Da)/pI	Theoretical MW (Da)/pI	Peptide Count	Score	Protein Score Confidence level (C.I.%)	Up/Down
201	ATP synthase CF1 alpha subunit (chloroplast)	*Lilium superbum*	YP_009130198.1	55,285.2/5.41	55,319.38/5.41	21	962	100	↑
259	ATP synthase alpha subunit, partial (mitochondrion)	*Erythronium dens-canis*	AFM91753.1	28,195.8/6.51	28,213.43/6.50	10	114	100	↑
202	ATP synthase CF1 alpha subunit (chloroplast)	*Lilium superbum*	YP_009130198.1	55,285.2/5.41	55,319.38/5.41	22	936	100	↑
908	Photosystem II oxygen evolving complex protein 2 precursor	*Fritillaria agrestis*	AAC04809.1	28,094.2/8.31	28,111.52/8.31	5	60	98.435	↓
284	ATP synthase CF1 beta subunit, partial (plastid)	*Lilium superbum*	AEZ48850.1	53,576.9/5.22	53,610.53/5.22	15	59	98.118	↑
294	Ribulose-1,5-bisphosphate carboxulase/oxygenase large subunit, partial (chloroplast)	*Heloniopsis kawanoi*	AIW53238.1	50,960.6/6.23	50,992.85/6.24	29	1180	100	↑
180	ATP synthase CF1 alpha subunit (chloroplast)	*Lilium superbum*	YP_009130198.1	55,285.2/5.41	5319.38/5.41	21	865	100	↑
896	Carbonic anhydrase	*Musa acuminata subsp.*	Tr|M0TL28	22,422.1/5.07	22,436.69/5.06	4	104	99.985	↑
716	PDZ domain-containing protein	*Cynara cardunculus var.*	Tr|A0A118JU51	36,198.3/6.18	36,220.78/6.18	6	113	99.998	↑
913	Chlorophyll a-b binding protein, chloroplastic	*Musa acuminata subsp.*	Tr|M0SBM9	29,718.2/8.96	29,737.07/8.96	4	171	100	↑
431	Actin	*Lilium davidii var. Davidii*	ALO18835.1	41,649.0/5.31	41,675.77/5.31	15	447	100	↑
511	Glutamine synthetase	*Tulipa pulchella*	BAM84282.1	38,673.4/5.64	38,697.60/5.64	5	90	99.999	↑
479	Actin	*Lilium regale*	AFU06383.1	41,619.0/5.31	41,645.75/5.31	16	421	100	↑
492	Monodehydroascorbate reductase	*Lilium longiflorum*	ADF43731.1	46,732.5/5.89	46,761.56/5.89	14	86	99.996	↑
220	ATP synthase CF1 alpha subunit (chloroplast)	*Lilium superbum*	YP_009130198.1	55,285.2/5.41	55,319.38/5.41	19	730	100	↓
988	Ribulose-1,5-bisphosphate carboxylase/oxygenase large subunit, partial (chloroplast)	*Gagea wilczekii*	AAM29162.1	50,739.4/5.96	50,771.69/5.96	12	494	100	↑
1060	Pathogenesis-related protein 10	*Lilium regale*	AHG94651.1	16,709.4/5.31	16,719.85/5.31	7	536	100	↓
136	Ribulose-1,5-bisphosphate carboxylase/oxygenase large subunit, partial (chloroplast)	*Trillium camschatcense*	AFP48691.1	44,763.6/6.52	44,792.01/6.53	9	63	99.14	↑
985	ATP synthase beta subunit, partial (chloroplast)	*Fritillaria acmopetala*	AKG96681.1	51,914.1/5.13	51,946.57/5.13	16	68	99.774	↓
142	ATP synthase CF1 alpha subunit (chloroplast)	*Ripogonum album*	ANO45506.1	55,341.1/5.26	55,375.27/5.26	10	72	99.91	↓
415	Glutamine synthetase	*Erythranthe guttata*	Tr|A0A022RZ30	39,028.6/5.40	39,053.05/5.39	7	393	100	↓
405	6-Phosphogluconate dehydrogenase, decarboxylating	*Citrus sinensis*	Tr|A0A067G3F9	53,519.6/6.38	53,553.34/6.38	13	437	100	↓
444	Elongation factor Tu	*Vigna angularis var. Angularis*	Tr|A0A0S3RGB1	52,659.2/6.34	52,692.26/6.34	14	671	100	↓
868	Chlorophyll a-b binding protein, chloroplastic	*Kalanchoe fedtschenkoi*	Tr|A0A089WZX0	28,226.3/5.15	28,244.20/5.15	7	195	100	↑
517	Glutamine synthetase	*Lolium perenne*	Tr|C5IW59	38,973.5/5.40	38,998.03/5.40	10	348	100	↓
866	Chlorophyll a-b binding protein, chloroplastic	*Carya cathayensis*	Tr|Q1KLZ3	28,296.3/5.15	28,314.25/5.15	5	101	99.969	↑
880	Beta carbonic anhydrase 3	*Arabidopsis thaliana*	Sp|Q9ZUC2	28,810.8/6.54	28,829.03/6.54	6	94	99.83	↑
864	Chlorophyll a-b binding protein, chloroplastic	*Kalanchoe fedtschenkoi*	Tr|A0A089WZX0	28,226.3/5.15	28,244.20/5.15	10	294	100	↓
860	Carbonic anhydrase	*Zea mays*	Tr|Q41729	71,291.9/8.93	71,337.55/8.93	10	101	99.969	↓
542	Ribulose bisphosphate carboxylase/oxygenase activase	*Medicago truncatula*	Tr|G7JTD2	52,135.9/5.42	52,169.06/5.42	15	358	100	↓
1037	Type II peroxiredoxin	*Medicago truncatula*	Tr|A0A072U4Q3	25,893.6/9.35	25,909.84/9.35	10	191	100	↓
304	ATP synthase CF1 beta subunit, partial (plastid)	*Lilium superbum*	AEZ48850.1	53,576.9/5.22	53,610.53/5.22	28	1190	100	↓
964	Ribulose-1,5-bisphosphate carboxylase/oxygenase large subunit, partial (chloroplast)	*Cardiocrinum giganteum var. Yunnanense*	AAM29161.1	50,201.1/6.04	50,233.05/6.04	12	88	99.998	↑
299	ATP synthase CF1 beta subunit, partial (plastid)	*Lilium superbum*	AEZ48850.1	53,576.9/5.22	53,610.53/5.22	21	112	100	↓
130	70 kDa heat shock protein	*Sandersonia aurantiaca*	AAL85887.1	36,768.6/4.83	36,791.57/4.82	3	260	100	↓
633	NADP-dependent alkenal double bond reductase P2	*Morus notabilis*	Tr|W9SE47	40,693.7/6.23	40,719.76/6.22	7	96	99.89	↓
90	Elongation factor G, mitochondrial	*Medicago truncatula*	Tr|A0A072UPP0	81,881.8/5.50	81,933.70/5.50	17	579	100	↓
671	Cysteine synthase	*Populus trichocarpa*	Tr|B9HJY5	34,176.2/7.64	34,197.70/7.64	11	132	100	↑
839	Putative L-ascorbate peroxidase 2, cytosolic-like	*Solanum chacoense*	Tr|A0A0V0HVQ3	28,638.6/5.75	28,656.76/5.75	9	324	100	↑
618	Trypsin-like serine protease	*Medicago truncatula*	Tr|G7KIR6	45,774.3/6.79	45,802.44/6.80	10	525	100	↑
182	FtsH-like protein Pftf	*Nicotiana tabacum*	Tr|Q9ZP50	74,335.8/6.00	74,382.14/6.00	24	658	100	↑
307	ATP synthase alpha subunit, partial (mitochondrion)	*Lilium lancifolium*	AAR28047.1	41,824.8/6.47	41,850.81/6.47	13	397	100	↑
313	Atpb (chloroplast)	*Lilium distichum*	AMT85217.1	53,546.9/5.22	53,580.50/5.22	17	113	100	↓
580	Glyceraldehyde-3-phosphate dehydrogenase, partial	*Lilium regale*	AHZ94971.1	36,779.2/7.11	36,802.04/7.11	12	255	100	↑
349	Ribulose-1,5-bisphosphate carboxylase/oxygenase large subunit, partial (chloroplast)	*Cardiocrinum giganteum var. Yunnanense*	AAM29161.1	50,201.1/6.04	50,233.05/6.04	19	159	100	↑
489	Monodehydroascorbate reductase	*Lilium longiflorum*	ADF43731.1	46,732.5/5.89	46,761.56/5.89	18	271	100	↓
752	Photosystem II oxygen evolving complex protein 1 precursor	*Fritillaria agrestis*	AAC04808.1	34,847.8/6.26	34,869.39/6.25	16	695	100	↓
263	Dihydrolipoyl dehydrogenase	*Salvia miltiorrhiza*	Tr|A0A0G2SJN7	53,520/6.96	53,553.72/6.96	6	259	100	↑
192	Malic enzyme	*Phaseolus angularis*	Tr|A0A0L9UG31	73,189.2/8.33	73,235.49/8.33	9	177	100	↓
604	Fructose-bisphosphate aldolase	*Oxytropis ochrocephala*	Tr|A0A0K1JSG5	42,894.2/6.39	42,920.76/6.39	9	477	100	↑

Note: Assigned spot number as indicated in Figure 3. Arrows indicate up- (↑) and down- (↓) regulation of the proteins.

## Supplementary Materials

Supplementary materials can be found at http://www.mdpi.com/1422-0067/19/12/3955/s1.

## Abbreviations

AtpA	ATP synthase CF1 alpha subunit (chloroplast)
Ci	intercellular CO_2_ concentration
DTT	DL-dithiothreitol
Fv/Fm	the maximum quantum yield of PSII
Gs	stomatal conductance
HRW	hydrogen-rich water
Pn	net photosynthetic rate
qP	photochemical quenching
SNP	sodium nitroprusside
TCA	trichloroacetic acid
Tr	transpiration rate
2-DE	two-dimensional electrophoresis
β-ME	β-mercaptoethanol
ФPSII	the effective quantum yield of PSII
