# Supplementary material for: Comparative Proteomic Analysis during the Involvement of Nitric Oxide in Hydrogen Gas-Improved Postharvest Freshness in Cut Lilies
Source: Int J Mol Sci. 2018 Dec 9;19(12):3955. doi: 10.3390/ijms19123955 (PMC6320913; doi:10.3390/ijms19123955)
Supplement: Supplementary file 1 [file ijms-19-03955-s001.pdf]

Supplementary Materials

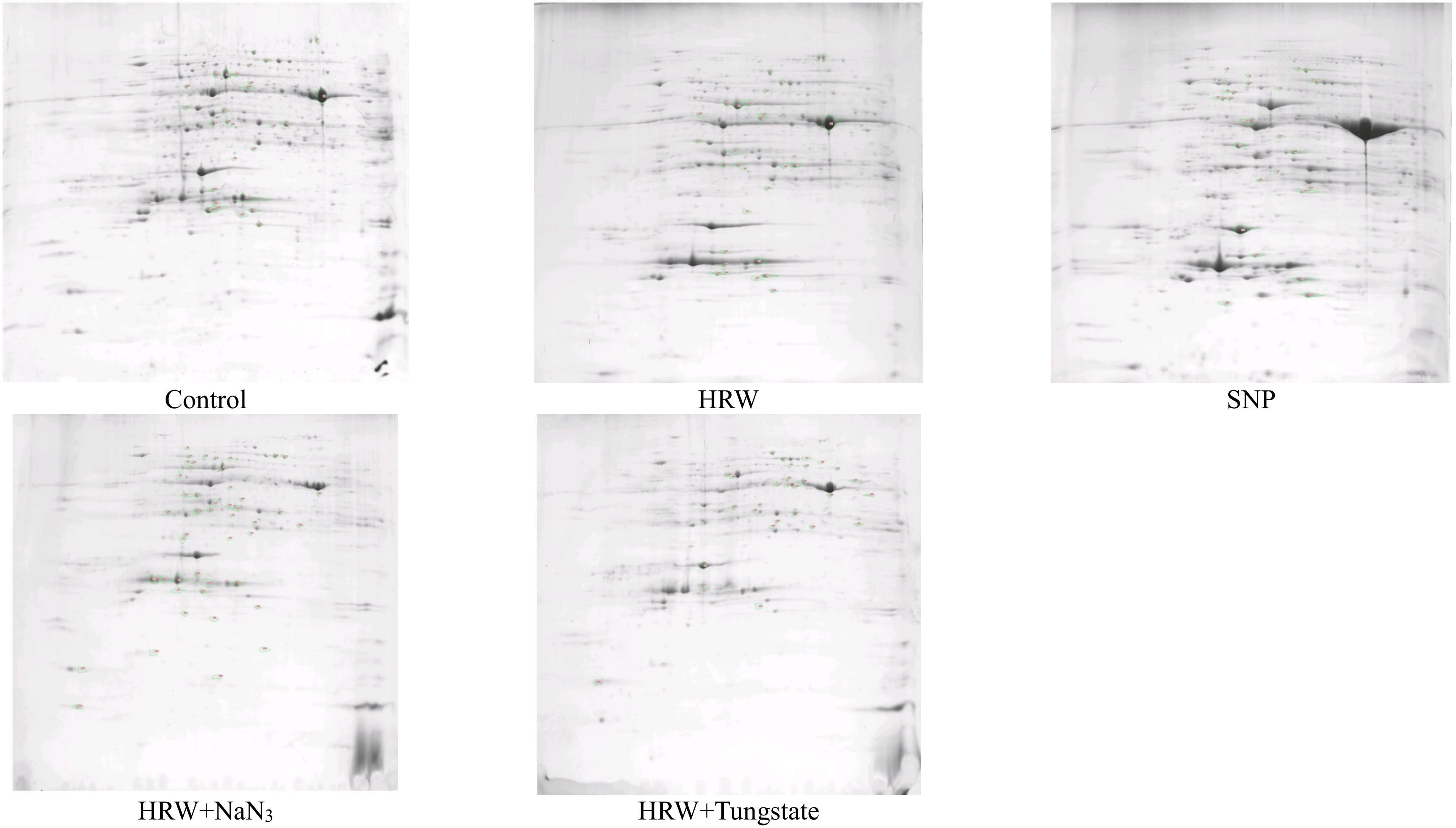

Figure S1. 2-DE gel images showed to employe protein spot densitometric analysis.

Table S1. The sequences of all unique peptides matched to each identified differential protein.

Spot No.	Protein name	Species	Pep. Count	Start Seq.	End Seq.	Sequences of unique peptides
201	ATP synthase CF1 alpha subunit (chloroplast)	*Lilium superbum*	21	166	172	ELIIGDR
				17	22	IEQYNR
				273	279	QMSLLLR
				406	413	ATQNQLAR
				120	128	GEISASESR
				6	14	ADEISNIIR
				15	22	ERIEQYNR
				481	491	TLTEQAEALLK
				129	140	LIESPAPGIISR
				108	119	VINALAKPIDGR
				177	188	TAVATDTILNQK
				256	266	HTLIIYDDLSK
				95	107	IAQIPVSEAYLGR
				255	266	RHTLIIYDDLSK
				468	480	NKPQFQEIISSTK
				285	297	EAYPGDVFYLHSR
				26	41	IVNTGTVLQVGDGIAR
				26	41	IVNTGTVLQVGDGIAR
				142	162	SVYEPLQTGLIAIDAMIPIGR
				385	405	LELAQFAELEAFAQFASD LDK
				141	162	RSVYEPLQTGLIAIDAMIPI
259	ATP synthase alpha subunit, partial (mitochondrion)	*Erythronium dens-canis*	10	110	119	AVDSLVPIGR
				87	97	VEVKAPGIIER
				134	145	TAIAIDTILNQK
				248	260	EAFPGDVFYLHSR
				49	64	TGSIVDVPAGKALLGR
				215	229	DNGMHALIIYDDLSK
				151	166	DVNPLYCVYVAIGQKR
				134	150	TAIAIDTILNQKQMNSR
				99	119	SVHEPMQTGLKAVDSLVPIG
				146	165	QMNSRDVNPLYCVYVAIGQK
202	ATP synthase CF1 alpha subunit (chloroplast)	*Lilium superbum*	22	166	172	ELIIGDR
				17	22	IEQYNR
				273	279	QMSLLLR
				406	413	ATQNQLAR
				120	128	GEISASESR
				6	14	ADEISNIIR
				15	22	ERIEQYNR
				481	491	TLTEQAEALLK
				129	140	LIESPAPGIISR
				108	119	VINALAKPIDGR
				177	188	TAVATDTILNQK
				256	266	HTLIIYDDLSK
				129	141	LIESPAPGIISRR
				95	107	IAQIPVSEAYLGR
				273	284	QMSLLLRRPPGR
				255	266	RHTLIIYDDLSK
				468	480	NKPQFQEIISSTK
				285	297	EAYPGDVFYLHSR
				26	41	IVNTGTVLQVGDGIAR
				26	41	IVNTGTVLQVGDGIARGR
				142	162	SVYEPLQTGLIAIDAMIPIGR
				141	162	RSVYEPLQTGLIAIDAMIPIGR
908	Photosystem II oxygen evolving complex protein 2 precursor	*Fritillaria agrestis*	5	117	126	EVEFPGQVLR
				234	244	LYICKAQAGDK
				27	42	LVPTIKPSQLICRAQK
				92	111	TNTDFLPYTGDGFNLLIPAK
				2	26	ASSACFLLHHAPSTPATTSRTPSHR
284	ATP synthase CF1 beta subunit, partial (plastid)	Lilium superbum	15	192	205	AHGGVSVFGGVGER
				487	498	AMNLEGESNLKK
				278	291	FVQAGSEVSALLGR
				249	261	VGLTALTMAEYFR
				232	246	VALVYGQMNEPPGAR
				3	18	INPTTSGLGVSTLEEK
				2	18	RINPTTSGLGVSTLEEK

				110<br>128<br>360<br>88<br>292<br>53<br>179	127<br>145<br>378<br>109<br>312<br>73<br>205	IFNVLGEPVDNLGPVDTR<br>TTSPIHRSAPAFIQLDTK<br>GIYPAVDPLDSTSTMLQPR<br>GMEVIDTGTPLSVPVGGATLGR<br>MPSAVGYQPTLSTEMGS LQER<br>DTVGPQINVTCEVQQLLGNNR<br>TVLIMELINNIAKAHGGVS
294	Ribulose-1,5-bisphosphate carboxulase/oxygenase large subunit, partial (chloroplast)	*Heloniopsis kawanoi*	29	301<br>183<br>301<br>291<br>28<br>417<br>299<br>281<br>435<br>213<br>335<br>301<br>17<br>130<br>197<br>142<br>160<br>211<br>446<br>142<br>446<br>232<br>330<br>232<br>335<br>190<br>17<br>254<br><br>37	307<br>189<br>307<br>298<br>36<br>426<br>307<br>290<br>445<br>222<br>345<br>311<br>27<br>141<br>208<br>154<br>172<br>222<br>458<br>156<br>460<br>247<br>345<br>248<br>351<br>208<br>36<br>280<br><br>74	NHGMHFR<br>AVYECLR<br>NHGMHFR<br>AMHAVIDR<br>DTDILAAFR<br>VALEACVQAR<br>QKNHGMHFR<br>DNGLLLHIHR<br>EGNEIIREASK<br>FLFCAEALYK<br>EMTLGFVDLLR<br>NHGMHFRVLAK<br>LTYYTPDYETK<br>LEDLRIPTSYSK<br>DDENVNSQPFMR<br>TFQGPPHGIQVER<br>YGRPLLGCTIKPK<br>DRFLFCAEALYK<br>WSPELAAACEVWK<br>TFQGPPHGIQVERDK<br>WSPELAAACEVWKEI<br>GHYLNATAGTCEEMIK<br>LEGEREMTLGFVDLLR<br>GHYLNATAGTCEEMIKR<br>EMTLGFVDLLRDDFIEK<br>GGLDFTKDDENVNSQPFMR<br>LTYYTPDYETKDTDILAAFR<br>ELGVPIVMHDYLTGGFTANTSLSHY CR<br>VTPQPGVPPEEAGAAVAAESSTGT WTTVWTDGLTSLDR
180	ATP synthase CF1 alpha subunit (chloroplast)	*Lilium superbum*	21	166<br>17<br>273<br>406<br>120<br>6<br>15<br>481<br>129<br>108<br>177<br>256<br>95<br>255<br>468<br>285<br>26<br>26<br>142<br>385<br>141	172<br>22<br>279<br>413<br>128<br>14<br>22<br>491<br>140<br>119<br>188<br>266<br>107<br>266<br>480<br>297<br>41<br>41<br>162<br>405<br>162	ELIIGDR<br>IEQYNR<br>QMSLLLR<br>ATQNQLAR<br>GEISASESR<br>ADEISNIIR<br>ERIEQYNR<br>TLTEQAEALLK<br>LIESPAPGIISR<br>VINALAKPIDGR<br>TAVATDTILNQK<br>HTLIIYDDLSK<br>IAQIPVSEAYLGR<br>RHTLIIYDDLSK<br>NKPQFQEIISSTK<br>EAYPGDVFYLHSR<br>IVNTGTVLQVGDGIAR<br>IVNTGTVLQVGDGIAR<br>SVYEPLQTGLIAIDAMIPIGR<br>LELAQFAELEAFAQFASD LDK<br>RSVYEPLQTGLIAIDAMIPI
896	Carbonic anhydrase	*Musa acuminata subsp.*	4	37<br>1<br>94<br>47	46<br>13<br>109<br>64	FLVFACADSR<br>MDPVERLTSGFER<br>VEYIVVIGHSRCGGIK<br>VCPSVVLSFQPGEAFIVR
716	PDZ domain-containing protein	*Cynara cardunculus var.*	6	152<br>160<br>288<br>199<br>298<br>297	157<br>169<br>297<br>209<br>312<br>312	TMYTIR<br>IGPLLMRMQK<br>SEEFEPLMKK<br>VREIQMQNYLR<br>YDESFINENALNAIK<br>KYDESFINENALNAIK

913	Chlorophyll a-b binding protein, chloroplastic	*Musa acuminata subsp.*	3	184<br>171<br>107	191<br>183<br>123	QYFLGLEK<br>RFQDWANPGSMGK<br>YAMLGAVGAIAPEIFGK
431	Actin	*Lilium davidii var. Davidii*	15	21<br>42<br>199<br>31<br>362<br>87<br>180<br>241<br>194<br>218<br>71<br>98<br>294<br>293<br>150	30<br>52<br>208<br>41<br>374<br>97<br>193<br>256<br>208<br>233<br>86<br>115<br>314<br>314<br>179	AGFAGDDAPR<br>HTGVMVGMGQK<br>GYMFTTTAER<br>AVFPSIVGRPR<br>AEFDESGPAIVHR<br>IWHHTFYNELR<br>LDLAGRDLTDSLMK<br>NYELPDGQVITIGAER<br>ILTERGYMFTTTAER<br>LAYVALDYEQELETAK<br>YPIEHGIVSNWDDMEK<br>VAPEEHPVLLTEAPLNPK<br>DLYGNIVLSGGSTMFPGIADR<br>KDLYGNIVLSGGSTMFPGIADR<br>TTGIVLDSGDGVSHTVPIYEGYALP HAILR
511	Glutamine synthetase	*Tulipa pulchella*	5	296<br>279<br>333<br>141<br><br>53	305<br>291<br>353<br>165<br><br>79	HETADINTFK<br>EHISAYGEGNERR<br>RPASNMDPYVVTSMIAETTLL<br>WPIGWPIGGYPGPQGPYYCSAGAD K<br>WNYDGSSTGQAPGEDSEVILYPQAI FK
479	Actin	*Lilium regale*	16	21<br>42<br>199<br>31<br>362<br>87<br>180<br>199<br>241<br>218<br>71<br>98<br>2<br>294<br>293<br>150	30<br>52<br>208<br>41<br>374<br>97<br>193<br>212<br>256<br>233<br>86<br>115<br>20<br>314<br>314<br>179	AGFAGDDAPR<br>HTGVMVGMGQK<br>GYMFTTTAER<br>AVFPSIVGRPR<br>AEFDESGPAIVHR<br>IWHHTFYNELR<br>LDLAGRDLTDSLMK<br>GYMFTTTAEREIVR<br>NYELPDGQVITIGAER<br>LAYVALDYEQELETAK<br>YPIEHGIVSNWDDMEK<br>VAPEEHPVLLTEAPLNPK<br>ADAEDIQPLVCDNGTGMVK<br>DLYGNIVLSGGSTMFPGIADR<br>KDLYGNIVLSGGSTMFPGIADR<br>TTGIVLDSGDGVSHTVPIYEGYALP HAILR
492	Monodehydroascorbat e reductase	*Lilium longiflorum*	14	305<br>53<br>311<br>77<br>422<br>40<br>8<br>63<br>166<br>287<br>202<br>249<br>413<br>184	310<br>62<br>319<br>85<br>433<br>52<br>22<br>76<br>183<br>304<br>218<br>269<br>433<br>201	LYNEIR<br>AYLFPQGAAR<br>RVEHVDHAR<br>LLPEWYTEK<br>QLITEGLNFASK<br>EAVAPYERPALSK<br>YIIIGGGVSAGYAAR<br>LPGFHVCVGSGGER<br>AVIVGGGYIGLELGATLK<br>TSITDVYAVGDVATFPLK<br>LFTAEIAAFYEGYYANK<br>VLDADIVVVGVGGRPLTTLFK<br>LQPAVADPKQLITEGLNFASK<br>INNLDVTMVYPEPWCMPR
220	ATP synthase CF1 alpha subunit (chloroplast)	*Lilium superbum*	19	166<br>17<br>406<br>6<br>15<br>481<br>129<br>108<br>177<br>256<br>191	172<br>22<br>413<br>14<br>22<br>491<br>140<br>119<br>188<br>266<br>202	ELIIGDR<br>IEQYNR<br>ATQNQLAR<br>ADEISNIIR<br>ERIEQYNR<br>TLTEQAEALLK<br>LIESPAPGIISR<br>VINALAKPIDGR<br>TAVATDTILNQK<br>HTLIIYDDLSK<br>NVICVYVAIGQK

				95<br>468<br>189<br>285<br>26<br>142<br>385<br>141	107<br>480<br>202<br>297<br>41<br>162<br>405<br>162	IAQIPVSEAYLGR<br>NKPQFQEIISSTK<br>GKNVICVYVAIGQK<br>EAYPGDVFYLHSR<br>IVNTGTVLQVGDGIAR<br>SVYEPLQTGLIAIDAMIPIGR<br>LELAQFAELEAFAQFASDLDK<br>RSVYEPLQTGLIAIDAMIPIGR
988	Ribulose-1,5-bisphosphate carboxylase/oxygenase large subunit, partial (chloroplast)	*Gagea wilczekii*	12	13<br>284<br>115<br>2<br>182<br>127<br>196<br>300<br>217<br>431<br>266<br>22	21<br>292<br>126<br>12<br>193<br>139<br>207<br>314<br>232<br>446<br>283<br>59	DTDILAAFR<br>QKNHGIHFR<br>LEDLRIPASYSK<br>LTYYTPDYETK<br>DDENVNSQPFMR<br>TFQGPPHGIQVER<br>DRFLFCAEAIYK<br>MSGGDHIHSGTVVGK<br>GHYLNATAGTCEEMMK<br>WSPELAAACEVWKEIK<br>DNGLLLHIHRAMHAVIDR<br>VTPQPGVPPEEAGAAVAAESSTGTWTTVWTDGLTSLDR
1060	Pathogenesis-related protein 10	*Lilium regale*	7	67<br>19<br>53<br>77<br>53<br>31<br>69	76<br>30<br>66<br>92<br>68<br>52<br>92	ERLDFVDHEK<br>AAILDWHTLGPK<br>QLNFTSVMPFSYVK<br>FECQSTIIEGGHLGTR<br>QLNFTSVMPFSYVKER<br>LVPEIISSGTGESGDGGAGSVR<br>LDFVDHEKFECQSTIIEGGHLGTR
136	Ribulose-1,5-bisphosphate carboxylase/oxygenase large subunit, partial (chloroplast)	*Trillium camschatcense*	9	1<br>8<br>279<br>122<br>191<br>212<br>212<br>315<br>295	7<br>16<br>287<br>134<br>202<br>227<br>228<br>331<br>314	TPDYTPK<br>DTDILAAFR<br>QKNHGIHFR<br>TFQGPPHGIQVER<br>DRFLFCAEALYK<br>GHYLNATAGTCEEMMK<br>GHYLNATAGTCEEMMKR<br>EMTLGFVDLLRDDVVLK<br>MSGGDHIHSGTVVGKLEGER
985	ATP synthase beta subunit, partial (chloroplast)	*Fritillaria acmopetala*	16	203<br>187<br>273<br>374<br>244<br>227<br>1<br>257<br>105<br>355<br>203<br>83<br>287<br>48<br>350<br>452	212<br>200<br>286<br>385<br>256<br>241<br>17<br>272<br>122<br>373<br>220<br>104<br>307<br>68<br>373<br>483	EGNDLYMEMK<br>AHGGVSVFGGVGER<br>FVQAGSEVSALLGR<br>IVGEEHYEIAQR<br>VGLTALTMAEYFR<br>VALVYGQMNEPPGAR<br>TTSGLGVSTLEEKNLGR<br>DVNEQDVLLFIDNIFR<br>IFNVLGEPVDNLGPVDTR<br>GIYPAVDPLDSTSTMLQPR<br>EGNDLYMEMKESGVINEK<br>GMEVIDTGTPLSVPVGGATLGR<br>MPSAVGYQPTLSTEMGSLQER<br>DTVGPQINVTCEVQQLLGNNR<br>GLAAKGIYPAVDPLDSTSTMLQPR<br>GFQLILSGELDSLPEQAFYLVGNIDEATAKAM
142	ATP synthase CF1 alpha subunit (chloroplast)	*Ripogonum album*	10	6<br>129<br>129<br>95<br>468<br>285<br>26<br>1<br>142<br>468	14<br>140<br>141<br>107<br>480<br>297<br>41<br>14<br>162<br>491	ADEISNIIR<br>LIESPAPGIISR<br>LIESPAPGIISRR<br>IAQIPVSEAYLGR<br>NKPQFQEIISSTK<br>EAYPGDVFYLHSR<br>IVNTGTVLQVGDGIAR<br>MVTLRADEISNIIR<br>SVYEPLQTGLIAIDSMIPIGR<br>NKPQFQEIISSTKTFTEQAEALLK
415	Glutamine synthetase	*Erythranthe guttata*	7	295<br>278<br>276<br>18	304<br>289<br>289<br>33	HETADINTFK<br>EHIAAYGEGNER<br>HKEHIAAYGEGNER<br>IIAEYVWIGGSGMDLR

				332	355	RPASNMDPYIVTSMIAETTILGKQ
				84	111	GNNILVMCDAYTPAGVPIPGNNRHNAAK
				52	78	WNYDGSSTGQAPGEDSEVILYPQAIFK
405	6-phosphogluconate dehydrogenase, decarboxylating	*Citrus sinensis*	13	26	34	GFPISVYNR
				353	361	SIEKGWDLK
				292	300	FLSGLKEER
				445	454	LPANLVQAQR
				455	464	DYFGAHTYER
				339	350	ICSYAQGMNLIR
				465	477	VDMSGSFHTEWFK
				339	352	ICSYAQGMNLIRAK
				79	96	AGSPVDQTIKTLSVYMEK
				97	113	GDCIIDGGNEWYENTER
				209	227	SVGKLSNEELQQVFSEWNK
				269	291	WTVQQAADLSVAAPTIESSLDAR
				416	441	VVCLAINSGISTPGMSSSLAYFDTYR
444	Elongation factor tu	*Vigna angularis var. Angularis*	14	323	333	VGETVDLVGLR
				472	483	TVGAGVIQSIIE
				121	131	YDEIDAAPEER
				120	131	KYDEIDAAPEER
				199	211	QVGVPNMVVFLNK
				319	333	GTIRVGETVDLVGLR
				349	364	ILDEALAGDNVGLLLR
				150	164	HYAHVDCPGHADYVK
				134	149	GITINTATVEYETENR
				83	99	KKPHVNIGTIGHVDHGK
				44	60	LTTLSSSFLQPSTVLRR
				100	119	TTLTAALTMALASLGNSA
				212	229	QDQVDDEELLQLVELEVR
				165	191	NMITGAAQMDGAILVVSGADGPMPQTK
868	Chlorophyll a-b binding protein, chloroplastic	*Kalanchoe fedtschenkoi*	7	98	105	ELEVIHSR
				127	134	FGEAVWFK
				96	105	NRELEVIHSR
				106	122	WAMLGALGCVFPELLSR
				38	56	TGAKPKPVSSGSPWYGPDR
				239	267	GPLENLADHLADPVNNNAWAYATNFVPGK
				59	95	YLGPFSGEAPSYLTGEFPGDYGWDTAGLSADPETFAK
517	Glutamine synthetase	*Lolium perenne*	10	279	290	EHIAAYGEGNER
				259	272	KDGGFEVIVAAVEK
				277	290	HKEHIAAYGEGNER
				19	34	IIAEYIWIGGSGMDLR
				1	18	MALLTDLLNLDLSGSTEK
				292	311	LTGKHETADIHTFSWGVANR
				296	316	HETADIHTFSWGVANRGASIR
				85	106	GNNILVMCDCYTPAGEPIPTNK
				85	107	GNNILVMCDCYTPAGEPIPTNKR
				53	79	WNYDGSSTGQAPGEDSEVILYPQAIFK
866	Chlorophyll a-b binding protein, chloroplastic	*Carya cathayensis*	5	98	105	ELEVIHSR
				127	134	FGEAVWFK
				38	56	TAGRPKPVSSGSPWYGPDR
				218	238	NGRLAMFSMFGFFVQAIVTGK
				239	267	GPLENLADHLADPVNNNAWAYATNFVPGK
880	Beta carbonic anhydrase 3	*Arabidopsis thaliana*	6	82	91	FLVFACADSR
				110	120	NIANMVPPYDK
				155	167	GLMAIEDNTAPTK
				77	91	SQTPKFLVFACADSR
				187	203	QDCKDLSFEDQCTNCEK
				232	250	GAHYDFVKGTFDLWELDFK
864	Chlorophyll a-b binding protein,	*Kalanchoe fedtschenkoi*	10	127	134	FGEAVWFK
				96	105	NRELEVIHSR

	chloroplastic			123	134	NGVKFGEAVWFK
				106	122	WAMLGALGCVFPELLSR
				106	122	WAMLGALGCVFPELLSR
				38	56	TGAKPKPVSSGSPWYGPDR
				221	238	LAMFSMFGFFVQAIVTGK
				239	267	GPLENLADHLADPVNNNAWAYATN
				178	212	FVPGK
						VAGGPLGEVVDPLYPGGSFDPLGL
				59	95	ADDPEAFAELK
						YLGPFSGEAPSYLTGEFPGDYGWD
						TAGLSADPETFAK
860	Carbonic anhydrase	*Zea mays*	10	474	482	KPELFGPLK
				27	39	LRPPGSGSSGTPR
				530	545	YTGIGSAIEYAVCALK
				311	326	AFTVRNIAAMVPGYDK
				528	545	TKYTGIGSAIEYAVCALK
				21	39	AVLLLRLRPPGSGSSGTPR
				392	408	KEHASVPFDDQCSILEK
				8	26	ATTSSIVPACHPRAVLLLR
				593	609	KEHASVPFDDQWSILEK
				165	185	DGAPDNFTFVEDWVRIGSPAK
542	Ribulose bisphosphate carboxylase/oxygenase activase	*Medicago truncatula*	15	354	360	VYDDEVR
				305	311	FYWAPTR
				174	183	SFQCELVFAK
				56	66	VSAKIDYNEEK
				305	314	FYWAPTRDDR
				361	373	NWISGIGVESIGK
				312	323	DDRVGVCQGIFR
				361	374	NWISGIGVESIGKR
				418	433	YLSEAALGDANDDAIK
				224	238	MCCLFINDLDAGAGR
				1	17	MATSVTIVGAINMAQLK
				335	351	IVDTFPGQSIDFFGALR
				222	238	GKMCCLFINDLDAGAGR
				280	298	VPIIVTGNDFSTLYAPLIR
				184	206	MGISPIMMSAGELESGNAGEPAK
1037	Type II peroxiredoxin	*Medicago truncatula*	10	214	222	STLVSNMHR
				185	195	YALLAEDGVVK
				40	52	FNSKPLRFSSSPK
				47	62	FSSSPKISATISVGDK
				10	25	LLSSSTTTSLLSPKTK
				214	228	STLVSNMHRQVMQTK
				164	181	AIGVELDLSDKPVGLGVR
				123	139	GIDTIACISVNDAFVMK
304	ATP synthase CF1 beta subunit, partial (plastid)	*Lilium superbum*	28	146	154	LSIFETGIK
				155	163	VVDLLAPYR
				135	145	SAPAFIQLDTK
				76	87	AVAMSATDGLMR
				192	205	AHGGVSVFGGVGER
				487	498	AMNLEGESNLKK
				278	291	FVQAGSEVSALLGR
				379	390	IVGEEHYEIAQR
				179	191	TVLIMELINNIAK
				206	217	TREGNDLYMEMK
				74	87	VRAVAMSATDGLMR
				232	246	VALVYGQMNEPPGAR
				23	39	IAQIIGPVLDVVFPPGK
				232	248	VALVYGQMNEPPGARMR
				1	18	MRINPTTSGLGVSTLEEK
				262	277	DVNEQDVLLFIDNIFR
				110	127	IFNVLGEPVDNLGPVDTR
				360	378	GIYPAVDPLDSTSTMLQPR
				88	109	GMEVIDTGTPLSVPVGGATLGR
				400	418	ELQDIIAILGLDELSEEDR
				53	73	DTVGPQINVTCEVQQLLGNNR
				292	312	MPSAVGYQPTLSTEMGS
				398	418	YKELQDIIAILGLDELSEEDR
				51	73	GRDTVGPQINVTCEVQQLLGNNR
				53	75	DTVGPQINVTCEVQQLLGNNRVR

				400<br>457<br><br>318	423<br>486<br><br>354	ELQDIIAILGLDELSEEDRLTVAR<br>GFQLILSGELDGLPEQAFYLVGNID EATAK<br>EGSITSIQAVYVPADDLTDPAPAATF AHLDATTVLSR
964	Ribulose-1,5-bisphosphate carboxylase/oxygenase large subunit, partial (chloroplast)	*Cardiocrinum giganteum var.*	12	13<br>444<br>198<br>420<br>2<br>164<br>127<br>300<br>1<br>217<br>427<br>22	21<br>451<br>207<br>430<br>12<br>174<br>139<br>314<br>12<br>232<br>443<br>59	DTDILAAFR<br>EIKFEFEP<br>FLFCAEAIYK<br>EGNEIIREACK<br>LTYYTPDYETK<br>NYGRAVYECLR<br>TFQGPPHGIQVER<br>MSGGDHIHSGTVVGK<br>KLTYYTPDYETK<br>GHYLNATAGTCEEMIK<br>EACKWSPELAAACEVWK<br>VTPQPGVPPEEAGAAVAAESSTGT WTTVWTDGLTSLDR
299	ATP synthase CF1 beta subunit, partial (plastid)	*Lilium superbum*	21	146<br>146<br>146<br>146<br>146<br>146<br>146<br>146<br>146<br>146<br>146<br>146<br>146<br>146<br>146<br>146<br>146<br>146<br>146<br>146<br>146	154<br>145<br>205<br>498<br>291<br>390<br>261<br>52<br>246<br>18<br>248<br>277<br>127<br>378<br>109<br>418<br>312<br>73<br>418<br>73<br>354	LSIFETGIK<br>SAPAFIQLDTK<br>AHGGVSVFGGVGER<br>AMNLEGESNLKK<br>FVQAGSEVSALLGR<br>IVGEEHYEIAQR<br>VGLTALTMAEYFR<br>MPNIYNALVVKGR<br>VALVYGQMNEPPGAR<br>INPTTSGLGVSTLEEK<br>VALVYGQMNEPPGARMR<br>DVNEQDVLLFIDNIFR<br>IFNVLGEPVDNLGPVDTR<br>GIYPAVDPLDSTSTMLQPR<br>GMEVIDTGTPLSVPVGGATLGR<br>ELQDIIAILGLDELSEEDR<br>MPSAVGYQPTLSTEMGSLQER<br>DTVGPQINVTCEVQQLLGNNR<br>YKELQDIIAILGLDELSEEDR<br>GRDTVGPQINVTCEVQQLLGNNR<br>EGSITSIQAVYVPADDLTDPAPAATF AHLDATTVLSR
130	70 kda heat shock protein	*Sandersonia aurantiaca*	3	234<br>20<br>119	244<br>36<br>141	NALENYAYNMR<br>MDKSTVHDVVLVGGSTR<br>EQVFSTYSDNQPGVLIQVYEGER
633	NADP-dependent alkenal double bond reductase P2	*Morus notabilis*	7	222<br>97<br>2<br>46<br>8<br>46<br>46<br>41	231<br>108<br>12<br>56<br>20<br>56<br>58<br>56	FGFDEAFNYK<br>TLQPITGYGVAK<br>AEVINKQVLLR<br>NLYLSCDPYMR<br>QVLLRDYVSGSAK<br>NLYLSCDPYMR<br>NLYLSCDPYMRGR<br>GVLVKNLYLSCDPYMR
90	Elongation factor G, mitochondrial	*Medicago truncatula*	17	265<br>554<br>513<br>725<br>582<br>31<br>413<br>551<br>142<br>330<br>159<br>506<br>627<br>690<br>122<br>525	276<br>566<br>524<br>737<br>595<br>42<br>429<br>566<br>158<br>348<br>176<br>524<br>646<br>709<br>141<br>547	AIVWGGEELGAK<br>VEANVGAPQVNYR<br>LAQEDPSFHFSR<br>VSHKTLSPCAYIK<br>QSGGQGQFADITVR<br>FMGTCFRPVQSR<br>VYSGKLTAGSYVLNSNK<br>EYKVEANVGAPQVNYR<br>GITITSAATTTFWDNHR<br>GSIAATFVPVMCGSAFKNK<br>INIIDTPGHVDFTLEVER<br>MAAGLVKLAQEDPSFHFSR<br>GLEECMSNGVLAGFPVVDVR<br>VEVVTPEEHLGDVIGDLNSR<br>IGEVHEGTATMDWMEQEQER<br>DEEINQTVIEGMGELHLEIIVDR
671	Cysteine synthase	*Populus*	11	293	304	LIVVVFPSFGER

		trichocarpa		159	170	VHYETTGPEIWK
				50	63	DRIGYSMIADAEAK
				265	282	EGLLVGISSGAAAAAAVK
				96	109	LIITMPASMSNERR
				35	49	IAAKLEGMEPCSSVK
				114	129	AFGAELVLTNPAKGMK
				93	108	GYKLIITMPASMSNER
				159	175	VHYETTGPEIWKGSGGK
				242	259	IVDEVVQISSDEAIETAK
				142	158	TPNAYILQQFENPSNPK
839	Putative L-ascorbate peroxidase 2, cytosolic-like	*Solanum chacoense*	9	31	38	NCAPIILR
				39	50	LAWHSAGTYDVK
				39	52	LAWHSAGTYDVKTK
				209	222	ALLEDPVFRPLVEK
				192	208	ELLSGEKEGLLQLPSDK
				68	85	HGANNGLDIAVRLLEPIK
				223	240	YAADEDAFFADYAEAHLK
				172	191	SGFEGAWTNNPLIFDNSYFK
				143	166	EVFGHMGLSDKDIVALSGGHTLGR
618	Trypsin-like serine protease	Medicago truncatula	10	325	333	VTRPILGIK
				419	428	EKIPVILEPK
				106	116	KLQTDELATVR
				158	169	DGHIVTNYHVIR
				188	201	VVGFDQDKDVAVLR
				209	225	LRPIPVGVSADLLVGQK
				207	225	DKLRPIPVGVSADLLVGQK
				226	248	VYAIGNPFGLDHTLTTGVISGLR
				135	157	QDAFTLDVLEVPQGSGSGFVWDK
				135	169	QDAFTLDVLEVPQGSGSGFVWDK DGHIVTNYHVIR
182	Ftsh-like protein pftf	*Nicotiana tabacum*	24	340	350	GTGIGGGNDER
				660	668	ETMTGDEFR
				101	109	FLEYLDKDR
				609	618	MMARNSMSEK
				338	350	QRGTGIGGGNDER
				241	251	QDFMEVVEFLK
				379	391	ADILDSALLRPGR
				241	251	QDFMEVVEFLK
				471	484	IVAGMEGTVMTDGK
				419	431	FDSDVSLEVIAMR
				457	470	TAIASKEIDDSIDR
				522	537	GLTWFIPADDPTLISK
				1	17	MATSSVCIAGNSLSTHR
				197	217	SPGGMGGPGGPGNPLAFGQSK
				1	18	MATSSVCIAGNSLSTHRR
				321	337	ENAPCIVFVDEIDAVGR
				630	646	LSDSAYEIALTHIRNNR
				319	337	AKENAPCIVFVDEIDAVGR
				432	453	TPGFSGADLANLLNEAAILAGR
				220	240	FQMEPNTGVTFDDVAGVDEAK
				113	134	VDLFENGTIAIVEAISPELGNR
				487	511	SLVAYHEVGHAICGTLTPGHDAVQK
				283	311	AIAGEAGVPFFSISGSEFVEMFVGV GASR
				351	378	EQTLNQLLTEMDGFEGNTGIIVVAA TNR
				580	608	QMVVTFGMSELGPWSLMDSSAQS GDVIMR
307	ATP synthase alpha subunit, partial (mitochondrion)	*Lilium lancifolium*	13	133	139	ELIIGDR
				87	94	GALSDHER
				361	367	LELAQYR
				120	129	AVDSLVPIGR
				75	86	VVDALGVPIDGR
				109	119	SVHEPMQTGLK
				144	155	TAIAIDTILNQK
				168	178	LYCVYVAIGQK
				329	342	GIRPAINVGLSVSR
				261	273	EAFPGDVFYLHSR
				228	242	DNGMHALIIYDDLSK

				9<br>368	28<br>389	VYGLNEIQAGEMVEFASGVK<br>EVAAFAQFGSDLDAATQALLNR
313	Atpb (chloroplast)	*Lilium distichum*	17	393<br>135<br>208<br>192<br>278<br>379<br>249<br>232<br>3<br>1<br>262<br>110<br>360<br>208<br>88<br>292<br>53	399<br>145<br>217<br>205<br>291<br>390<br>261<br>246<br>18<br>18<br>277<br>127<br>378<br>225<br>109<br>312<br>73	QTSQRYK<br>SAPAFIQLDTK<br>EGNDLYMEMK<br>AHGGVSVFGGVGER<br>FVQAGSEVSALLGR<br>IVGEEHYEIAQR<br>VGLTALTMAEYFR<br>VALVYGQMNEPPGAR<br>INPTTSGLGVSALEEK<br>MRINPTTSGLGVSALEEK<br>DVNEQDVLLFIDNIFR<br>IFNVLGEPVDNLGPVDTR<br>GIYPAVDPLDSTSTMLQPR<br>EGNDLYMEMKESGVINEK<br>GMEVIDTGTPLSVPVGGATLGR<br>MPSAVGYQPTLSTEMGSLQER<br>DTVGPQINVTCEVQQLLGNNR
580	Glyceraldehyde-3-phosphate dehydrogenase, partial	*Lilium regale*	12	114<br>261<br>260<br>209<br>56<br>332<br>182<br>33<br>32<br>243<br>318<br>280	125<br>271<br>271<br>223<br>68<br>344<br>195<br>48<br>48<br>259<br>331<br>295	EGAGKHIQAGAK<br>TFAEEVNAAFR<br>KTFAEEVNAAFR<br>AAALNIVPTSTGAAK<br>YDSTLGIFGADVK<br>VVDLADIVANNWK<br>GTMTTTHSYTGDQR<br>DSPLEIIAINDTGGVK<br>KDSPLEIIAINDTGGVK<br>VPTPNVSVVDLVVQVSK<br>VIAWYDNEWGYSQR<br>GILSVCDEPLVSVDFR
349	Ribulose-1,5-bisphosphate carboxylase/oxygenase large subunit, partial (chloroplast)	*Cardiocrinum giganteumvar. Yunnanense*	19	168<br>13<br>402<br>284<br>266<br>420<br>320<br>2<br>182<br>127<br>145<br>431<br>431<br>217<br>427<br>320<br>175<br>239<br>22	174<br>21<br>411<br>292<br>275<br>430<br>330<br>12<br>193<br>139<br>157<br>443<br>446<br>233<br>443<br>336<br>193<br>265<br>59	AVYECLR<br>DTDILAAFR<br>VALEACVQAR<br>QKNHGMHFR<br>DNGLLLHIHR<br>EGNEIIREACK<br>EMTLGFVDLLR<br>LTYYTPDYETK<br>DDENVNSQPFMR<br>TFQGPPHGIQVER<br>YGRPLLGCTIKPK<br>WSPELAAACEVWK<br>WSPELAAACEVWKEIK<br>GHYLNATAGTCEEMIKR<br>EACKWSPELAAACEVWK<br>EMTLGFVDLLRDDFIEK<br>GGLDFTKDDENVNSQPFMR<br>ELGVPIVMHDYLTGGFTANTSLSHYCR<br>VTPQPGVPPEEAGAAVAAESSTGTWTTVWTDGLTSLDR
489	Monodehydroascorbate reductase	*Lilium longiflorum*	18	305<br>53<br>311<br>77<br>27<br>86<br>422<br>40<br>8<br>63<br>391<br>166<br>225<br>287<br>202<br>249<br>184	310<br>62<br>319<br>85<br>39<br>97<br>433<br>52<br>22<br>76<br>405<br>183<br>243<br>304<br>218<br>269<br>201	LYNEIR<br>AYLFPQGAAR<br>RVEHVDHAR<br>LLPEWYTEK<br>QGLAPGELAIISK<br>GIELLLSTEIVK<br>QLITEGLNFASK<br>EAVAPYERPALSK<br>YIIIGGGVSAGYAAR<br>LPGFHVCVGSGGER<br>VVGAFLESGSPDENK<br>AVIVGGGYIGLELGATLK<br>GTVAVGFDSDANGDVTAVK<br>TSITDVYAVGDVATFPLK<br>LFTAEIAAFYEGYYANK<br>VLDADIVVVGVGGRPLTTLFK<br>INNLDVTMVYPEPWCMPR

				413	433	LQPAVADPKQLITEGLNF
752	Photosystem II oxygen evolving complex protein 1 precursor	*Fritillaria agrestis*	16	235<br>206<br>89<br>320<br>88<br>235<br>262<br>245<br>243<br>189<br>33<br>214<br>164<br>245<br><br>289<br>189	242<br>213<br>97<br>329<br>97<br>242<br>273<br>261<br>261<br>205<br>53<br>234<br>184<br>269<br><br>313<br>213	GSSFLDPK<br>VPFLFTVK<br>LTFDEIQSK<br>IQGIWYAQLE<br>RLTFDEIQSK<br>GSSFLDPK<br>GDEEELVKENIK<br>GGSAGYDNAVALPAGGR<br>GRGGSAGYDNAVALPAGGR<br>DGIDYAAVTVQLPGGER<br>AFGFDNSTAGRLTCSINSDLR<br>QLVATGKPESFSGSYLVPSYR<br>LTYTLDEIEGPFEVAPDGTVK<br>GGSAGYDNAVALPAGGRGDEEELV<br>K<br>SKPETGEVIGVFESIQPSDTDLGSK<br>DGIDYAAVTVQLPGGERVPFLFTVK
263	Dihydrolipoyl dehydrogenase	*Salvia miltiorrhiza*	6	417<br>495<br>482<br>92<br>76<br>290	427<br>507<br>494<br>104<br>91<br>314	VGKFPLLANSR<br>EAAMATYDKPIHI<br>TCHAHPTMSEALK<br>ALLHSSHMYHEAK<br>GALGGTCLNVGCIPSK<br>LTIEPAAGGEQTTLEADVVLVSAGR
192	Malic enzyme	*Phaseolus angularis*	9	135<br>606<br>252<br>180<br>480<br>630<br>648<br>480<br>2	143<br>616<br>263<br>189<br>494<br>643<br>662<br>498<br>20	GLAFTEGER<br>GLAFTEGER<br>GLAFTEGER<br>GLAFTEGER<br>AIKPTVLIGSSGVGK<br>AYDLGLASHLPRPK<br>YAESCMYSPGYRSYR<br>AIKPTVLIGSSGVGKTFTK<br>SHSSGCDVSKTLGLCICER
604	604	Fructose-bis phosphate aldolase	9	240<br>204<br>174<br>349<br>282<br>84<br>69<br>83<br>151	246<br>211<br>183<br>358<br>293<br>96<br>82<br>96<br>173	TFEVAQK<br>EAAWGLAR<br>TAAYYQQGAR<br>TWGGRPENVK<br>ATPEQVADYTLK<br>LASIGLENTEANR<br>GILAMDESNATCGK<br>RLASIGLENTEANR<br>GLVPLAGSNDESWCQGLDGLASR
